# Overview of single‐cell RNA sequencing analysis and its application to spermatogenesis research

**DOI:** 10.1002/rmb2.12502

**Published:** 2023-01-28

**Authors:** Takahiro Suzuki

**Affiliations:** ^1^ RIKEN Center for Integrated Medical Science (IMS) Yokohama City Kanagawa Japan; ^2^ Graduate School of Medical Life Science Yokohama City University Yokohama City Kanagawa Japan

**Keywords:** next‐generation sequencing, single‐cell analysis, single‐cell RNA sequencing, spermatogenesis, testis

## Abstract

**Background:**

Single‐cell transcriptomics allows parallel analysis of multiple cell types in tissues. Because testes comprise somatic cells and germ cells at various stages of spermatogenesis, single‐cell RNA sequencing is a powerful tool for investigating the complex process of spermatogenesis. However, single‐cell RNA sequencing analysis needs extensive knowledge of experimental technologies and bioinformatics, making it difficult for many, particularly experimental biologists and clinicians, to use it.

**Methods:**

Aiming to make single‐cell RNA sequencing analysis familiar, this review article presents an overview of experimental and computational methods for single‐cell RNA sequencing analysis with a history of transcriptomics. In addition, combining the PubMed search and manual curation, this review also provides a summary of recent novel insights into human and mouse spermatogenesis obtained using single‐cell RNA sequencing analyses.

**Main Findings:**

Single‐cell RNA sequencing identified mesenchymal cells and type II innate lymphoid cells as novel testicular cell types in the adult mouse testes, as well as detailed subtypes of germ cells. This review outlines recent discoveries into germ cell development and subtypes, somatic cell development, and cell–cell interactions.

**Conclusion:**

The findings on spermatogenesis obtained using single‐cell RNA sequencing may contribute to a deeper understanding of spermatogenesis and provide new directions for male fertility therapy.

## 1 INTRODUCTION

For over 20 years since the human genome project, transcriptome analysis has been dramatically developed, supported by the development of next‐generation sequencers and the growth of computer technologies. Recently, the resolution of transcriptome analysis has reached the single‐cell level. The single‐cell transcriptome analysis, such as single‐cell RNA sequencing (scRNA‐seq), can provide novel insights into the complex biological system in which many types of cells are involved, such as spermatogenesis. On the other hand, scRNA‐seq analysis is composed of highly specialized skills in experimental molecular biology and bioinformatics, making people hesitate to approach it. In the first section, I overview the development of transcriptome analysis and describe basic knowledge of the widely accepted spermatogenesis.

### 1.1 Gonadal development

**FIGURE 1:**
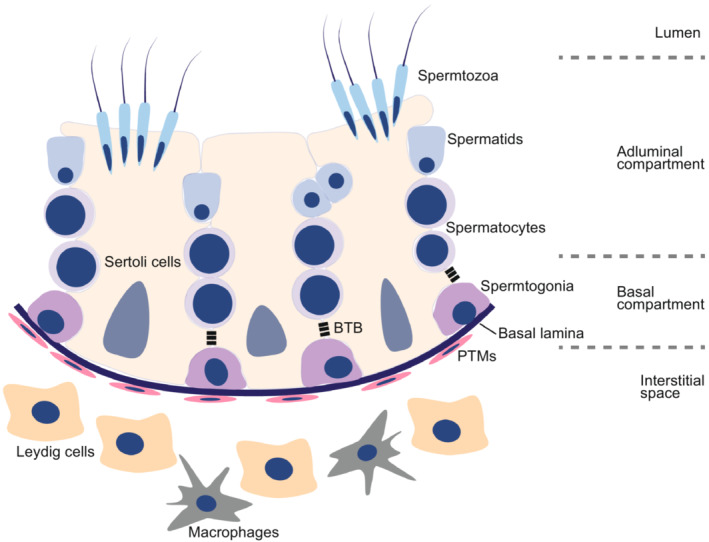
Testicular cells and spermatogenesis. Spermatogenesis occurs within seminiferous tubules. Spermatogonia, undifferentiated male gem cells in the testis, are located in the basal compartment. Along with spermatogenesis, spermatocytes, differentiated from spermatogonia, move through the blood‐testis barrier (BTB) of Sertoli cells, somatic cells located in seminiferous tubules, and enter the adluminal compartment, which is regulated by multiple signaling pathways such as TGF‐β/Smad, and MAPK signaling pathways. Then, the spermatocytes undergo two meiotic divisions, resulting in round haploid spermatids, elongated spermatids, and spermatozoa. Spermatozoa are released into the lumen of the seminiferous tubules. Other somatic cells, including Lydig cells, and macrophages, are located in interstitial space. Peritubular myoid cells (PTMs) surround the outside of seminiferous tubules.

The testes are male reproductive glands that support lifelong spermatogenesis. They first emerge as genital primordia, a common ancestor of the testes and ovaries, from the mesonephros on approximately embryonic day (E) 10 in mice and at 4 weeks of embryonic age in humans.^1^ After E10.5 in mice, primordial germ cells (PGCs) migrate to gonadal primordia, which is differentiated from proximal epiblast cells upon bone morphogenetic protein (BMP) stimulation at around E6.5.^2^ In the male gonadal primordium, SRY, a transcription factor encoded by a gene on the Y chromosome, emerges at around E11.5‐12.0 in mice and 6 weeks of embryonic age in humans and activates the downstream genes necessary for testis development,^3^, ^4^, ^5^, ^6^, ^7^, ^8^ which determine the developmental fate of the genital primordium to testes. After the sex determination, a specialized structure of the testes forms (Figure 1). The main testis component is seminiferous tubules that compose most of the testis. The inside of seminiferous tubules is lined with Sertoli cells as the epithelial layer, and spermatogenesis takes place at the boundaries between adjacent Sertoli cells, and spermatozoa are released from the epithelial layer to the testicular lumen. Other testicular somatic cells, including Leydig cells, peritubular myoid cells (PTMs), and immune cells are located at the interstitial space of a testis. Paracrine factors secreted from these testicular somatic cells and endocrine factors support the proper spermatogenesis, closely communicating with each other. In addition, migrated PGCs differentiate into prospermatogonia (ProSPG) (also called prespermatogonia or testicular gonocytes) in male fetal testes, along with sex determination.^2^ ProSPG is initially mitotically active (M‐ProSPG) and then progress to primary transitional (T1)‐ProSPG, which are located at the center of the seminiferous tubule and are mitotically quiescent. Soon after birth, T1‐ProSPG migrate to the basal lamina side of the seminiferous tubule and convert to secondary transitional (T2)‐ProSPG, which initiate proliferation.^9^ Then, some ProSPG differentiate directly to differentiating SPG and initiate spermatogenesis, skipping undifferentiated SPG stage, known as the first wave of spermatogenesis.^10^ Other ProSPG on the other hand, become SSCs for lifelong steady‐state spermatogenesis.

### 1.2 Spermatogenesis

Spermatogenesis, a multistep process that generates mature sperm from spermatogonial stem cells (SSCs), takes place in the seminiferous tubules generally after puberty (steady‐state spermatogenesis; Figure 1). On the other hand, unlike the postpuberty steady‐state spermatogenesis, the first wave of spermatogenesis, which occurs soon after birth in rodents but not in humans, appears to originate from nonself‐renewing SPG or directly from ProSPG but not from the self‐renewing SSCs.^9^ SSCs are a subset of spermatogonia (SPG) and maintain their pool by mitotic self‐renewal and can also give rise to source cells for spermatogenesis via asymmetric cell division.

In mice, SSCs are considered identical or included in A‐single spermatogonia, a subset of undifferentiated SPG. A‐single spermatogonia give rise to two A‐paired spermatogonia with an incomplete cell division that does not complete cytokinesis, interconnected via intercellular bridges to form a syncytium. Further incomplete cell divisions cause 4–16 cells of undifferentiated SPG (A‐align spermatogonia). Undifferentiated SPG are then divided into approximately 512 cells as differentiating SPG (Type A1, A2, A3, A4, In, and B SPG).^11^, ^12^ In contrast, human undifferentiated SPG increase to four cells with two incomplete cell divisions. Only one cell incomplete division occurs during the differentiating SPG stage, generating eight cells, which are interconnected by intercellular bridges.^11^ After mitotic divisions at the SPG stage, differentiating SPG convert into spermatocytes, initiating meiosis.^13^, ^14^ Meiosis prophase I is subdivided into leptotene, zygote, pachytene, diplotene, and diakinesis stages. Then, the cells undergo two meiotic divisions, resulting in round haploid spermatids, which undergo a morphological change, known as spermiogenesis, to form elongated spermatids and spermatozoa.^13^, ^14^ Finally, spermatozoa are released into the lumen of the seminiferous tubules. The germ cells from one SSC remain connected by cytoplasmic bridges until elongated spermatids.^15^, ^16^, ^17^

### 1.3 Testicular somatic cells

Sertoli cells are the “nurse cells” and the only somatic cell type in the seminiferous tubules, which directly contact germ cells in the seminiferous tubules. Sertoli cells are responsible for many critical functions for spermatogenesis. At the neonatal stage, Sertoli cells regulate the migration of gonocytes to the basement membrane of seminiferous tubules and stimulate their proliferation to establish the SSC pool.^18^, ^19^, ^20^ Sertoli cells also construct a crucial structure, blood‐testis‐barrier (BTB), which is specialized junctions between adjacent Sertoli cells composed of adherens, gap, and tight junctions. The BTB physically separates the abluminal compartment and lumen of seminiferous tubules from the basal compartment and interstitial space, maintaining the immune‐privileged environment. Spermatogonial cell division and differentiation occur in the basal compartment, whereas miosis and postmiotic development occur in the abluminal compartment. Thus, spermatocytes must move through the BTB to enter meiosis, which is regulated by multiple signaling pathways such as TGF‐β/Smad, and MAPK signaling pathways.^21^ Leydig cells in the interstitial space are mainly responsible for steroidogenesis, such as testosterone, upon luteinizing hormone (LH) stimulation. Testosterone regulates PTMs, Leydig, and Sertoli cells through the androgen receptor.^22^ Leydig cells are also sources of other humoral factors essential for the proliferation of spermatogonial stem cells (SSCs), such as GDNF, CSF1, and IGF1.^23^, ^24^, ^25^, ^26^ Seminiferous tubules are surrounded by PTMs, which mainly function in seminiferous tubule contraction to transport spermatozoa. PTMs also play an essential role in maintaining SSCs by secreting glial cell line‐derived neurotrophic factor upon testosterone‐androgen receptor binding.^27^, ^28^, ^29^ In addition to the major testicular somatic cells, immune cells, such as macrophages and natural killer cells, are known to reside in the interstitial space. Two subtypes of macrophages are identified in the interstitial space, one of which lines on the surface of seminiferous tubules, and the other is associated with vasculatures.^30^, ^31^, ^32^ The macrophages located around the vascular are associated with vascular development and promoting steroidogenesis of Leydig cells.^33^, ^34^, ^35^ On the other hand, peritubular macrophages contribute to spermatogonial differentiation, potentially through CSF1 and retinoic acid signaling.^30^, ^31^ Thus, interstitial cells influence germinal stem cell self‐renewal and differentiation, forming microenvironments suitable for each cell status, referred to as stem cell niche. However, although stem cell niches of model organisms, such as *Drosophila* and *C. elegans*, are spatially defined, those of mammals appear to be randomly located along the seminiferous tubules.^36^

### 1.4 History of transcriptomics

In the 1990s, genome and cDNA projects were launched for many species, including humans and mice.^37^, ^38^, ^39^, ^40^, ^41^ These projects have allowed researchers to analyze biological phenomena and diseases on a genome‐wide level. These genome‐wide comprehensive analyses, including genomic, transcriptomic, and epigenomic analyses, are known as omics analyses. Among omics analyses, transcriptomics, in which whole transcripts of a sample are obtained and analyzed, is the most powerful tool to delineate the overall picture of biological phenomena at the molecular level. The initial stage of transcriptomics utilized expressed sequence tags (EST).^42^ EST represents sequences of several hundred bases from the 5′‐ or 3′‐ends of the cDNA clones in a library, roughly reflecting transcript abundance in the library. Because one sequencing reaction in a standard sanger sequencer typically yields one EST, EST analysis needs many sequencing reactions to obtain enough quantitativity.

On the other hand, serial analysis of gene expression (SAGE),^43^, ^44^, ^45^ cap analysis of gene expression (CAGE),^46^ and their derivatives sequence concatenated short fragments (11–27 bp) from the 3′‐ or 5′‐ends of the cDNAs, increasing number of the sequencing tags in one sequencing reaction. These fragments are then computationally mapped to the genome and counted by gene, thus theoretically reflecting the gene expression profile. While 3′‐end sequencing methods such as SAGE are robust to incomplete reverse transcription of cDNA synthesis, 5′‐end sequencing methods such as CAGE provide exact transcription start site information, which is important to identify the gene regulatory regions.

Unlike sequencing‐based transcriptome technologies, microarrays have been widely used for transcriptome analysis. A microarray is a glass slide or silicon wafer on which oligonucleotides designed to hybridize to the cDNA of targets are spotted. cDNAs from cells or tissues of interest are labeled with fluorescent dyes and captured by the microarray spots. Microarrays have been widely used because of their cost efficiency and technical convenience. However, because the length of the oligonucleotide probes on most of the microarrays is about 20–60‐mer, which targets a tiny part of the transcript, the microarray loses the information on RNA processing events. Furthermore, because the microarray probes are designed for the known transcripts, microarray cannot identify the novel transcript.

Thanks to the emergence of next‐generation sequencers (NGSs) in 2006 and subsequent developments, transcriptome analysis has evolved extensively, overcoming the drawbacks of sequencing capacity and cost‐effectiveness of the former‐generation sequencers. Similar to SAGE and CAGE, RNA sequencing (RNA‐seq), an NGS‐based transcriptomic technology, reads the sequences of all transcripts in a library and counts the sequencing reads mapped to known and unknown transcripts. On the other hand, because RNA‐seq typically provides the full‐length sequence of transcripts, RNA‐seq provides information on RNA processing events. If random primers are used for the reverse transcription for cDNA synthesis but not oligo dT primers, RNA‐seq also captures nonpoly(A) transcripts such as long noncoding RNAs. Along with the decreasing of NGS sequencing cost, RNA‐seq became one of the current standard methods for transcriptome analysis.

## 2 TECHNICAL OVERVIEW OF SINGLE‐CELL RNA SEQUENCING

**FIGURE 2:**
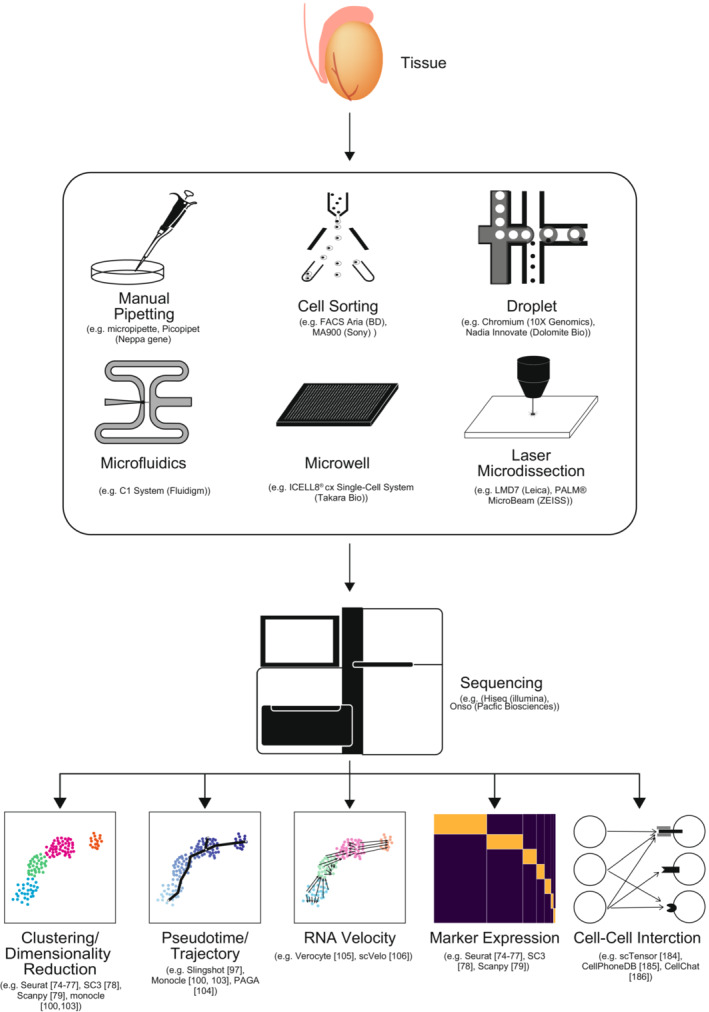
Workflow of single‐cell RNA‐seq analysis. Tissue is dissociated and subjected to single‐cell capturing. Single cells are captured by either manual pipetting, cell sorting, droplet, microfluidics, microwell, or laser microdissection. Representative systems for each cell capturing system are shown. A scRNA‐seq library is constructed and sequenced using next‐generation sequencing. Bioinformatic analyses, such as clustering, dimensionality reduction, pseudotime/trajectory, RNA velocity, marker expression, and cell–cell interaction analyses, are performed using scRNA‐seq. Several examples of R or Python packages for each analysis are shown. The numbers in brackets are reference numbers of each package.

Canonical transcriptomics typically analyzes RNA from a pool of cells, referred to as bulk transcriptome analyses. In the case of heterogeneous cell populations, such as tissues, the bulk transcriptome analyses provide the average transcriptome of all composing cells. Because bulk transcriptome analyses do not show the composition of the cell population, the result of bulk transcriptome analyses is a linear combination of the transcriptome of each cell population, losing the information of gene expression change of each cell population. On the other hand, newly developed scRNA‐seq technologies provide transcriptomes at the single‐cell level, overcoming the shortcomings of bulk transcriptome analyses (Figure 2).

### 2.1 Single‐cell RNA sequencing library construction

The first step in scRNA‐seq is cell dissociation to release individual cells. The standard cell dissociation method, a traditional method used for many applications, such as flow cytometry, uses tissue dissociation enzymes, including trypsin and collagenase, at 37°C. Because the distinct characteristics of different cell types and tissue types, such as stiffness and size, affect cell dissociation, this step may damage and deplete specific cell types, significantly affecting the outcome of scRNA‐seq data.^47^ Therefore, the cell dissociation protocol should be carefully optimized for the specific tissue type. In addition, a recent report suggested that this dissociation method induces a stress response at the gene expression level, affecting the scRNA‐seq results.^48^, ^49^ On the other hand, cell dissociation on ice, which can avoid the stress response occurring during cell dissociation at 37°C, using a cold‐active protease, provides a better approach for scRNA‐seq.^49^

After the cell dissociation, several approaches can be applied to capture single cells (Figure 2). For the analysis targeting a relatively low number of cells (~several hundred cells), dissociated individual cells are captured by manual pipetting from cell suspension or cell sorting and are isolated to individual wells of 96‐ or 384‐well microtiter plate. Microfluidic systems, such as Fluidigm® C1 platforms, are also used to capture the individual cell.^50^, ^51^ Laser microdissection, which directly captures single cells from a tissue section without cell dissociation, is another option for single‐cell capture.^52^ In contrast, microwell‐ and droplet‐based approaches are used to analyze more than 1000 cells.^51^ Because microwell‐ and droplet‐based approaches require specialized instruments, commercialized systems are provided, including the ICELL8® cx Single‐Cell system from Takara Bio, the Nadia Innovate system from Dolomite Bio, and the Chromium system from 10X® Genomics. In particular, the Chromium system is a currently widely used platform.^53^ While microwell and droplet‐based approaches require specialized instruments, the split‐pool combination barcoding method (known as SPLiT‐Seq) can analyze tons of single cells without customized equipment, lowering the hurdles of the high‐throughput scRNA‐seq.^54^

The captured single cells are then subjected to RNA‐seq library construction. The sequencing libraries are either full‐length, 3′‐end, or 5′‐end cDNAs. Full‐length cDNA libraries such as SMART‐seq® methods^55^, ^56^, ^57^, ^58^, ^59^ detect RNA processing events, such as alternative and aberrant RNA splicing.^60^ While SMART‐seq® methods use dT primer for the reverse transcription, the random displacement amplification sequencing (RamDA‐seq®) utilizes random primer, detecting nonpoly‐A transcripts, such as noncoding RNA.^61^ Because of the small amount of RNA from a single cell, cDNA amplification is typically required for scRNA‐seq library preparation. PCR is typically utilized for cDNA amplification, but PCR unevenly amplifies the cDNA due to the difference of length, GC content, and stochasticity. Therefore, a large part of scRNA‐seq protocols including MARS‐seq,^62^ Drop‐seq,^63^ inDrop,^64^ CEL‐seq2,^65^ SCRB‐seq,^66^ Quartz‐seq2,^67^ SMART‐seq3,^56^ and FLASH‐seq^59^ adopt a unique molecular identifier (UMI) to ensure quantifiability.^68^, ^69^ UMI is a random sequence tag incorporated into the sequencing library before PCR amplification. Theoretically, every single molecule has a UMI. Therefore, the amplification bias can be compensated by counting unique UMI sequences. Thus, many scRNA‐seq library construction methods were established. Representative methods were benchmarked in several reports, providing clues to select an optimal library construction method for each experiment.^70^, ^71^, ^72^

### 2.2 Computational analysis of scRNA‐seq data

As well as bulk RNA‐seq, the scRNA‐seq library is sequenced using a short read sequencer such as Hiseq (Illumina) (Figure 2). Then, combining bioinformatic techniques, the comprehensive parallel transcriptome data generated using scRNA‐seq provides many biological aspects (Figure 2). Currently, several useful integrated scRNA‐seq analysis packages, such as Seurat and Scanpy are provided as R or Python packages, which provide typical workflow of scRNA‐seq analysis.^73^, ^74^, ^75^, ^76^, ^77^, ^78^ scRNA‐seq data are first subjected to dimensionality reduction and cell clustering to overview the interrelation of all single cells. Every single cell is defined by its gene expression profile, which means the number of parameters to define the cell status equals the number of genes. Genes are not independently regulated but regulated as groups. Each gene group is regulated by the same upstream factor, such as the transcription factor, resulting in a highly correlated expression profile. Therefore, a much smaller number of parameters is enough to capture the all single‐cell status. Dimensionality reduction simplifies the parameters to 2–3, enabling the cell to be projected onto a 2‐ or 3‐dimensional space. Principal component analysis (PCA), diffusion maps,^79^ t‐distributed stochastic neighbor embedding (t‐SNE),^80^ uniform manifold approximation and projection (UMAP),^81^, ^82^ and reversed graph embeddings, such as DDRTree,^83^ are typically used for dimensionality reduction in scRNA‐seq analyses.

Principal component analysis computes a smaller number of uncorrelated variables as “principal components (PCs).” These resulting PCs are used for downstream analysis, such as clustering. However, because more than two PCs are generally required to capture the cell status in scRNA‐seq analysis, further dimensionality reduction, such as t‐SNE or UMAP, is needed for the visualization. t‐SNE compresses so that points that are close to a higher dimensional space are also close to the compressed lower dimensional space. UMAP carries out a similar points compression to t‐SNE but is lower computational cost by optimization of the different cost functions.

Unsupervised clustering is a process of aggregating single cells into several groups based on gene expression similarity without any training data. The cell grouping (cluster) typically represents the cell types. For the unsupervised clustering for scRNA‐seq data, diverse algorithms are used, including graph‐based methods,^63^, ^78^ *k*‐means clustering,^77^, ^84^ and hierarchical clustering.^85^, ^86^ Because the distinct clustering algorisms generate different grouping, careful consideration is required for result interpretation.^87^ The known marker expression patterns should corroborate the clustering results and predict the cell type. Machine‐learning approaches, which are based on training data, have also been proposed for cell‐type prediction.^88^, ^89^, ^90^, ^91^ Based on the clustering results, conventional transcriptome analyses, such as differential gene expression analysis, gene ontology (GO) enrichment, and pathway enrichment analyses, are performed for downstream analyses between clusters in a sample or between the same clusters of different samples. GO enrichment analysis identify overrepresented GO terms, which is standardized term of gene features such as function and localization. In these comparative analyses, cells in a cluster can be considered “biological replicates.” However, other analyses, such as cell composition and pseudotime/trajectory analyses, may need biological replicates of the target sample. Therefore, although the cost of scRNA‐seq is currently very high, taking biological replicates is recommended.^92^, ^93^

In addition to conventional analyses, characteristics analyses of scRNA‐seq data are also employed to discover novel insights. In particular, pseudotime/trajectory analysis is one of the unique analyses of scRNA‐seq.^83^, ^94^, ^95^, ^96^, ^97^, ^98^, ^99^, ^100^, ^101^ Pseudotime/trajectory analysis predicts the cellular differentiation path by aligning cells on a trajectory based on similarity. Most pseudotime/trajectory algorithms are based on graph‐based approaches, such as the minimum spanning tree^95^, ^100^ and partition‐based graph abstraction,^101^ following to the dimensionality reduction. The pseudotime of every single cell is inferred based on the trajectory, manually defining one side as the start of the time course. It should be noted that the algorithm must be selected based on the study design because the inferable trajectory types, such as linear, cycle, and branch, are different.

Pseudotime/trajectory analyses are intended in one direction. However, cellular differentiation has been proposed to be reversible in some instances. RNA velocity analysis is another method to infer cellular differentiation path that predict a cell's differentiation vector.^102^, ^103^ Usually, reads sequenced in scRNA‐seq are mapped and counted based on exons of known transcripts; however, scRNA‐seq reads also include intronic sequences derived from nascent (unspliced) transcripts. Since nascent transcripts eventually become mature transcripts by splicing, the expression profile of nascent transcripts predicts the cells' future status. Thus, compared with the mature transcript expression profiles of other cells, RNA velocity vectors: magnitude, and direction are defined for each cell, predicting the future fate of each cell.

Thus, although the quantifiability of scRNA‐seq is lower than that of bulk RNA‐sequencing because of the lower sequence depth per cell, scRNA‐seq is a powerful tool for analyzing complex tissues. Recently, because the testes consist of many cell types and germ cells at different differentiation stages, scRNA‐seq has been used to investigate spermatogenesis. The following sections provide a summary of novel insights into spermatogenesis recently reported using single‐cell transcriptomics.

## 3 SINGLE‐CELL TRANSCRIPTOMICS IN SPERMATOGENESIS

The number of reports about single‐cell transcriptomics in spermatogenesis is progressively increased after 2018. As of December, 18, 2022, a PubMed search with the words “spermatogenesis”, “single cell”, and “RNA sequencing” identified 58 original articles, excluding review, comment, or data descriptor articles. Of 58 articles, 46 were about humans or mice. Thus, currently most of the scRNA‐seq for spermatogenesis research has been done in humans or mice and more reports are required to discuss other species such as goats and rats. Therefore, based on the PubMed search and manual curation, I summarize recent novel findings about human and mouse spermatogenesis in this section.

### 3.1 Discovery of novel testicular cell types

scRNA‐seq can identify cell types that have not been previously identified. Using scRNA‐seq of whole testes of adult C57BL/6J mice, transcription factor 21 (TCF21)+ mesenchymal cells were identified as a novel testicular somatic cell type.^104^ Rank correlation analysis revealed that the TCF21+ mesenchymal cell population is reminiscent of an embryonic interstitial cell progenitor.^104^ scRNA‐seq of human testes showed that embryonic interstitial cell progenitors, which are potential counter parts of mouse TCF21+ mesenchymal cells, give rise to multiple testicular somatic cell types, such as PTMs and Leydig cells.^104^, ^105^ Thus, these scRNA‐seq analyses suggest that the TCF21+ mesenchymal cell population includes stem Leydig cells, a progenitor of adult Leydig cells.^106^ Although whether PTMs have mesonephric or gonadal cell origin remains controversial,^107^ these scRNA‐seq analyses support that PTMs are derived from gonadal interstitial progenitor cells. Consistent with the mouse scRNA‐seq analyses, human Leydig and myeloid cells were suggested to be derived from the same progenitor population.^108^

scRNA‐seq of perinatal and adult whole C57BL/6 mouse testes also identified innate lymphocytes as a novel testicular somatic cell type.^104^, ^109^ Marker expression patterns suggested that testis‐resident innate lymphocytes are type II (ILC2) innate lymphoid cells.^104^ ILC2 cells have been identified in various tissues, such as the lung, intestinal mucosa, and adipose tissue. ILC2 cells are known to be involved in immune system responses, such as allergies and protection from parasite infection.^110^ However, the function of ILC2 cells in the testis are yet to be determined. Because testicular macrophage plays essential roles in spermatogenesis, ILC2 cells may also contribute to spermatogenesis.

### 3.2 Novel subpopulation of prospermatogonia during its development

**FIGURE 3:**
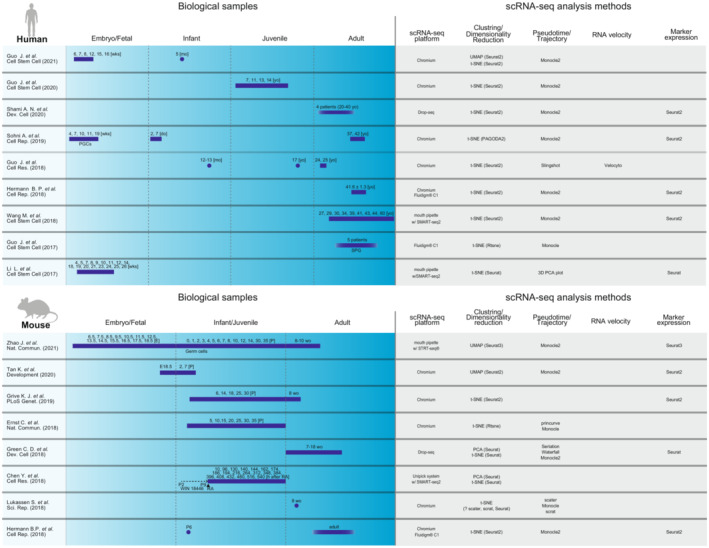
Human and mouse spermatogenesis single‐cell RNAseq analysis datasets. The graphical illustration represents the biological sample collection (left) and methods (right) of each scRNA‐seq analysis. The blue bars are the age range of samples. Rows represent each article. Human datasets are divided into embryo/fetal (before birth), infant (postnatal to 13 months old), juvenile (13 months old to 20 years old), and adult (20 years old+) stages. Mouse datasets are divided into embryo/fetal (before birth), infant/juvenile (postnatal to 35‐days postpartum), and adult (after 35‐days postpartum) stages. wks, do, wo, mo, yo, E, and P represent weeks of pregnancy, days old, weeks old, months old, years old, embryonic day, and postnatal day, respectively. In the dataset of Chen. Y. et al. Sci. Rep. (2018), P2 mice were treated with WIN 18446 until P9, and then retinoic acid (RA) was administrated to synchronize spermatogenesis. The scRNA‐seq platforms and packages for the analysis are shown in right side.

Germ cell development and differentiation are the main processes of spermatogenesis. To date, classification of spermatogenic cells mostly relies on histology, morphology, and marker protein expression. Recently, taking advantage of scRNA‐seq, which can analyze multiple germ cell states in parallel, germ cell development and spermatogenesis have been delineated in humans and mice, covering all developmental stages from PGC to spermatid (Figure 3).^104^, ^108^, ^111^, ^112^, ^113^, ^114^, ^115^, ^116^, ^117^, ^118^, ^119^, ^120^, ^121^ Using scRNA‐seq, Tan et al.^109^ identified a novel distinct ProSPG subtype, intermediate (I)‐ProSPG in mice. Transcriptome analysis suggested that the status of I‐ProSPG is between that of T1‐ and T2‐ProSPG and that I‐ProSPG specifically expresses *Elmo1* and *Palld*. Histological analysis revealed that I‐ProSPG are mainly located at the center of seminiferous tubules and are mitotically inactive. However, a few mitotically active I‐ProSPGs (ELMO1+ PALLD+) were identified at the periphery of the seminiferous tubules. Thus, I‐ProSPG appears to be a migratory intermediate between T1‐ and T2‐proSPG. Another independent scRNA‐seq study in mice also detected a ProSPG subtype similar to I‐ProSPG, corroborating the existence of I‐ProSPG.^117^

Human fetal or neonatal male germ cells are not as well characterized as those of mice. Sohni et al. described germ cell subsets by investigating human neonate testes and male fetal germ cells.^111^ They identified PGC‐like cells (PGCL) and two pre‐SPG populations (preSPG1 and preSPG2) using unsupervised clustering and trajectory/pseudotime analyses. Combining previously published transcriptome data of embryonic PGCs,^122^ they proposed that human germ cells gradually develop from PGC (embryo) to PGCL (neonate), to pre‐SPG (neonate), and finally to SSC (adult). Although further analyses are needed, the proposed male germ cell development trajectory suggests that human pre‐SPG corresponds to mouse ProSPG.

### 3.3 Subtypes in the spermatogonial stem cell population

Because SSCs can potentially recover the fertility of childhood cancer survivors by transplantation, it must be important to identify specific markers to isolate SSCs.^123^ SSCs are recognized to express specific prototypical SSC markers, such as ID4, GFRA1, and NANOS3. However, a growing body of evidence indicates the heterogeneity of SSCs.^124^, ^125^, ^126^, ^127^, ^128^, ^129^, ^130^, ^131^, ^132^, ^133^, ^134^

In mice, single‐cell qRT‐PCR analysis suggested that even the ID4+ SPG population is heterogeneous and the ID4^bright^ SPG population is an SSC‐enriched population.^135^, ^136^ scRNA‐seq studies further characterized the ID4^bright^ SPG population. In addition to the prototypical SSC markers, such as *Gfra1* and *Id4*, scRNA‐seq identified novel specific markers of the ID4^bright^ SPG population, including *Dusp6*, *Epha2*, *Ptpn13*, *Pvr*, *Tcl1*, *Cd82*, and *Lhx1*,^113^ which may be helpful to isolate primitive SSCs.^109^ Notably, pseudotime/trajectory analysis identified a subset in the ID4^bright^ SPG population, corresponding to a more primitive SSC subtype in adult testes. The more primitive SSC subtype specifically expresses genes involved in the hepatic stellate cell activation pathway (*Bcl2*, *Ednra*, *Klf6*, *Pdgfra*, and *Tgfa*) before expressing prototypical SSC markers in humans and mice, suggesting the signal to maintain SSC quiescence.^113^ On the other hand, an immature testis‐specific subtype of the ID4^bright^ SPG population featured the expression of genes involved in cell cycle regulation, proliferation, and morphogenesis.^113^ In summary, scRNA‐seq analyses suggested the primitive SSC subtype in the ID4^bright^ SPG population and transition from immature proliferative SSC to adult quiescent SSC.

**FIGURE 4:**
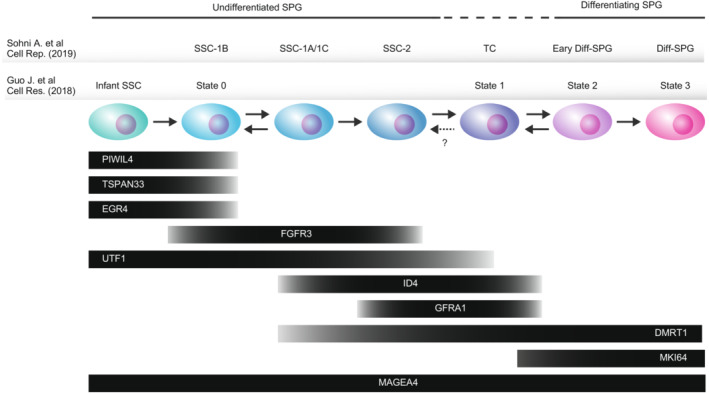
Summary of human spermatogonial differentiation. The scRNA‐seq analyses from Sohni et al. Cell Rep. (2019) and Guo et al. Cell Res. (2018) are summarized. Definitions of subsets in each report are shown at the upper. Arrows represent differentiation paths. The dotted arrow is the differentiation path, which is not completely evident. Marker expression patterns that were consistently reported in both studies are shown at the bottom. SPG, spermatogonia; SSC, spermatogonial stem cells; TC, transition cells.

Although understanding human SSCs is important for treating infertility, human SSCs are not well understood because human SSC‐to‐spermatogonia differentiation is largely different from that of mice. In humans, SSCs appear to be morphologically recognized as the Adark undifferentiated spermatogonia subtype. Using scRNA‐seq, Guo et al. analyzed SSEA4+ undifferentiated SPG and Kit+ differentiating SPG sorted from the adult human testes.^115^ The authors identified four distinct transcriptional states (states 1 to 4) of SPG, and SSEA4+ cells predominantly belonged to state 1, indicating an SSC population (Figure 4). They also analyzed human testes from infants (12–13 months old) and young adults (17, 24, and 25 years old) using scRNA‐seq in an independent report.^112^ In addition to the four spermatogonial states identified in the earlier report, the authors identified state 0. Pseudotime/trajectory analysis suggested that state 0 is the most undifferentiated state, resembling infants' earliest/naïve SSC^112^, ^121^ (Figure 4). Both state 0 and 1 populations express ID4, indicating heterogeneity in ID4+ cell populations, similar to mouse SSCs. Interestingly, RNA velocity analysis showed long vectors from state 2 to state 1, suggesting the reversibility of SPG, which is consistent with previous reports^137^, ^138^, ^139^ (Figure 4). A total of 490 genes were identified that were highly or explicitly expressed in state 0, including PIWL4, EGR4, TSPAN33, PHGDH, PPP1R36, and ICAL.^112^ However, state 0 cells did not express GFRA1, a prototypical SSC marker, suggesting that the state 0 cells are the same SSC subpopulation reported by Grisanti et al.^133^

Sohni et al.^111^ also reported novel subtypes of SSC and SPG in the scRNA‐seq analysis of adult human testes. They identified four SPG states based on unsupervised clustering, namely, SSC‐1, SSC‐2, early differentiating SPG, and differentiating SPG. Re‐clustering and pseudotime/trajectory analyses further segregated SSC‐1 into three subtypes (SSC‐1A, SSC‐1B, and SSC‐1C), and they proposed that SSC‐1B is the most primitive SSC status (Figure 4). Notably, they identified novel specific markers for the most primitive SSC, including PIWIL4, LPPR3, and TSPN33, which highly overlapped with the state 0 markers reported by Gou et al.^112^ suggesting that the SSC‐1B population is identical to state 0 SSC (Figure 4).

They also identified novel subtypes of SSC and differentiated SPG in humans.^111^ Transition cells (TCs), a distinct cell subset in human adult testes, appear to represent the transition status between undifferentiated and differentiating SPG.^111^ Supporting the nature of TCs, and the status between infrequent and active cell proliferation, TCs specifically express CCND2 and SPRY1, cell proliferation‐promoting proteins.^140^, ^141^

Together, analysis of scRNA‐seq data revealed the trajectory of spermatogonial development (Figure 4) and consistently reported a primitive SSC population, which can potentially be applied to treat male infertility. Importantly, because a particular primitive SSC marker is yet to be established in humans, surface proteins, such as LPPR3 and TSPN33, are helpful in enriching the most primitive SSCs.

### 3.4 The behavior of sex chromosome‐linked genes

During male meiosis, asynapsis of sex chromosomes induces meiotic sex chromosome inactivation (MSCI), forming an XY body.^142^, ^143^, ^144^, ^145^, ^146^, ^147^, ^148^ These inactivated sex chromosome‐linked genes are partially reactivated during spermiogenesis, but another sex chromosome inactivation occurs because of postmeiotic sex chromatin (PMSC) formation during the late spermatid stage.^149^ The series of sex chromosome‐linked gene regulations was quantitatively and temporally confirmed using scRNA‐seq.^104^, ^112^, ^113^, ^114^, ^118^, ^119^, ^120^, ^147^ The sex chromosome‐linked genes are highly expressed in SPG, and their expression dramatically decreases during meiosis, followed by reactivation after meiosis. Although the precise timing of MSCI is controversial, scRNA‐seq analyses suggested that MSCI occurs during meiosis prophase I in both mice and humans.^114^, ^119^, ^147^ Interestingly, although the timing and silencing degree of MSCI is similar between X‐ and Y chromosome‐linked genes, Y chromosome‐linked gene silencing appears to occur before X chromosome‐linked gene silencing, and the silencing degree is more intense than X chromosome‐linked genes in PMSC, suggesting that transcripts from Y‐linked genes are more unstable.^120^, ^147^

Haploid spermatids have either X or Y chromosomes as a result of meiosis. Several studies have suggested that X and Y chromosome‐bearing haploid spermatids from the same parent cell share transcripts via intercellular bridges^150^, ^151^, ^152^; however, extensive analysis of transcript sharing has not been performed. Taking advantage of the comprehensive parallel analysis, scRNA‐seq studies have investigated transcript sharing between X and Y chromosome‐bearing haploid spermatids in mice.^104^, ^119^ These reports show no apparent bias in X and Y chromosome‐linked gene expression among genetically distinct haploid spermatids, indicating global transcript sharing.

### 3.5 Testicular somatic cell development

**FIGURE 5:**
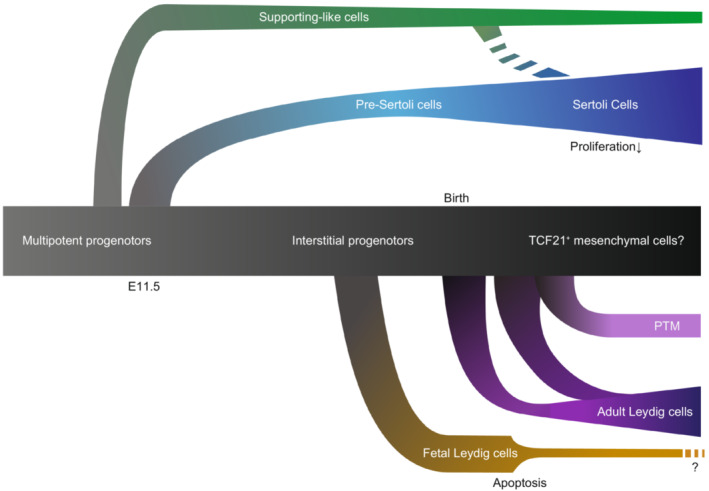
Summary of mouse testicular somatic cell development. Multipotent progenitors in a gonadal primordium give rise to supporting‐like cells before the emergence of pre‐Sertoli cells and supporting‐like cells are suggested to differentiate Sertoli cells. Pre‐Sertoli cells eventually differentiate into Sertoli cells after birth. The multipotent progenitors become interstitial progenitors after E11.5 and give rise to fetal Leydig cells. After birth, the interstitial progenitors appear to become transcription factor 21 (TCF21)^+^ mesenchymal cells, which differentiate into both peritubular myoid cells (PTMs) and adult Leydig cells. Whether fetal Leydig cells are maintained at the adult stage is controversial.

Testicular somatic cell development and detailed characteristics have also been determined using scRNA‐seq in mouse testes. Before sex determination, gonadal primordia are mainly composed of somatic multipotent precursor cells that are explicitly expressing Nr5a1, Wt1, Gata4,^153^, ^154^, ^155^, ^156^, ^157^ and Numb.^158^ A study using lineage tracing showed that both fetal Sertoli and Leydig cells are derived from WT1+ progenitor cells during embryogenesis.^159^ scRNA‐seq analyses corroborated the Sertoli and Leydig developmental courses at the transcriptome level using fetal mouse gonads. Trajectory/pseudotime analysis in mice revealed that some Nr5a1+ multipotent progenitors give rise to pre‐Sertoli cells at approximately E10.5‐E11.5, the timing of sex determination and that pre‐Sertoli cells mature during embryogenesis.^154^ Consistent with a previous report,^160^ the proliferation activity of mouse Sertoli cells declined after birth, and the transcriptomes between P2 and P7 were dramatically changed, suggesting a transition from immature to mature Sertoli cells^109^ (Figure 5). The transition from mitotically active immature Sertoli cells to mitotically inactive mature Sertoli cells was observed around puberty (11–14 years old) in human scRNA‐seq.^108^ A recent scRNA‐seq study in mouse embryonic gonads identified a previously uncharacterized population of supporting‐like cells (SLCs).^161^ SLCs originated from the Nr5a1+ multipotent progenitors emerging from E10.5. Similar to pre‐Sertoli cells, SLCs co‐express somatic cell markers such as *Wt1* and *Nr5a1*; Sertoli cell markers such as *Gata4* and *Sox9*. In addition, SLCs characteristically express *Pax8*. Although authors suggested that SLCs are also a distinct source of Sertoli cells from pre‐Sertoli cells, further independent studies are needed to validate the possibility (Figure 5).

After the E11.5 mouse embryo, the location of somatic progenitor cells is limited to the interstitial compartment of testicular tissue. It has been suggested that fetal Leydig cells differentiate from the interstitial progenitors.^154^ Fetal Leydig cells are believed to be gradually replaced by functionally distinct adult Leydig cells after birth^162^ but several reports have claimed that a small population of fetal Leydig cells persists in the postnatal stage in mice and rats.^163^, ^164^, ^165^, ^166^, ^167^ scRNA‐seq analysis by Ernst et al.^147^ showed that most fetal Leydig cells were replaced by adult Leydig cells by P15 in mice. Tan et al.^109^ also suggested that murine postnatal Leydig cells differ from prenatal Leydig cells. Thus, scRNA‐seq did not detect persistent fetal Leydig cells after birth. The fate of fetal Leydig cells in postnatal testes requires more careful analysis, which may be available by reusing published scRNA‐seq datasets.

Combined with the finding that TCF21^+^ mesenchymal cells seem to be the common precursors of Leydig and PTMs and that they may be derived from interstitial precursors, somatic cell development is summarized in Figure 5.

### 3.6 Seminiferous tubule cycle

Spermatogenesis is orchestrated by periodically cycling through the 12 stages of the seminiferous epithelial cycle (I to XII). The seminiferous epithelial cycle is canonically defined by the distinct set of germ cell types observed in the seminiferous tubule cross‐section.^168^, ^169^, ^170^ In mice, each seminiferous epithelial cycle takes 8.6 days, and the entire term of spermatogenesis takes ~35 days.^171^ In stages VII and VIII, SPG initiates spermatogenesis through retinoic acid exposure.^172^ Sertoli cells play a central role in the seminiferous epithelial cycle, showing different sizes and marker gene expression patterns at each stage.^173^, ^174^, ^175^, ^176^ Because the adult Sertoli cell is large (larger than 300 μm^3^ in mice), droplet‐based scRNA‐seq for adult testes frequently depleted Sertoli cells. Therefore, enrichment of Sertoli cells before scRNA‐seq using transgenic mice, such as SOX9‐GFP transgenic mice, is applied to obtain a sufficient number of adult Sertoli cells for scRNA‐seq analysis.^104^, ^177^ These scRNA‐seq analyses segregated adult Sertoli cells into several clusters, which correspond to seminiferous epithelial stages, indicating differential transcriptomes between stages. Interestingly, stages VI‐VIII were consistently clearly segregated as a cluster, suggesting that Sertoli cells at the stages of retinoic acid pulse show noticeable transcriptome characteristics.

### 3.7 Cell–cell interaction

Cells in a tissue intercommunicate through direct contact, gap junctions, and paracrine signaling using hormonal factors. Taking advantage of the parallel transcriptome data of all cell types in a tissue, scRNA‐seq data predict the cell–cell interactions based on the expression pattern of receptors and corresponding ligand pairs.^178^, ^179^, ^180^ For example, although previous reports illustrated that Notch signaling in mouse Sertoli cells promotes the differentiation of quiescent proSPG,^181^, ^182^ scRNA‐seq revealed that receptors of Notch signaling, such as *Notch2* and *Notch3*, are expressed in nearly all testicular somatic cell types, including Sertoli, Leydig, PTMs, and endothelial cells.^108^, ^109^, ^111^, ^112^, ^116^, ^117^ Ligands of Notch signaling, such as DLL1, DLL2, DLL4, and DLK1, are also expressed in almost all testicular cell types, including somatic and germ cells.^108^, ^109^, ^111^, ^112^, ^116^ Thus, Notch signaling plays a broader role in spermatogenesis than is currently known.

Activin signaling is essential in Sertoli cell differentiation and function.^183^, ^184^, ^185^ Ligands of activin signaling, activin, and inhibin, have been reported to be expressed in Sertoli cells, Leydig cells, PTMs, and germ cells.^186^, ^187^ scRNA‐seq analyses confirmed that activin/inhibin is expressed in stromal, PTMs, Sertoli, and Leydig cells.^108^, ^109^, ^112^ On the other hand, activin receptor genes (*Acvr1b*, *Bmpr1b*, and *Acvr2b*) are expressed in SPG, whereas their inhibitor genes, *Fst*, *Bambi*, and *Nog* are selectively expressed in undifferentiated SPG. Thus, activin signaling appears to play an essential role in spermatogonial differentiation.^108^

In addition to the Notch and activin signaling pathways, other pathways for ligand‐receptor pairs, including retinoic acid, kit, Gndf, Wnt, Fgf, and Hedgehog pathways, have been suggested to be involved in spermatogenesis by scRNA‐seq. Thus, these novel findings of cell–cell interactions obtained by scRNA‐seq data should be further validated.

### 3.8 Pathological analysis using scRNA‐seq

scRNA‐seq is also helpful for pathological analysis, especially in human biopsy samples. Nonobstructive azoospermia (NOA) is a major pathological cause of male infertility. Wang et al. compared scRNA‐seq data from a patient with NOA and healthy testes and identified a panel of differentially expressed genes in Sertoli cells.^114^ Since the identified differentially expressed genes were largely involved in spermatogenesis, these genes are potential drug targets, although only one NOA patient was tested. Zhao et al.^188^ also observed a substantial difference between normal and NOA Sertoli cells. Furthermore, they reported differences among NOA types (idiopathic NOA, Klinefelter syndrome, and Y chromosome AZF region microdeletion). Importantly, using pathway analysis of idiopathic NOA and normal adult Sertoli cells, Wnt signaling was found to be a possible drug target for idiopathic NOA therapy.

## 4 FUTURE PERSPECTIVES

Due to the development and application of NGS, substantial transcriptome data can be obtained at a relatively low cost. Accordingly, many transcriptome datasets are available in public data repositories, and the number of datasets is increasing daily. Because transcriptome data, in particular scRNA‐seq, are comprehensive, the dataset can be reused to analyze facets of biological questions other than those included in the original analysis. Furthermore, combinatorial analysis of multiple datasets obtained from independent studies may also provide new insights. In addition, because one of the primary roles of transcriptome analyses is to provide fundamental material for hypothesis building, showing the overall description of the biological phenomena, further experimental validations based on the transcriptome data‐derived hypothesis are essential.

scRNA‐seq methods are still being developed. For instance, current scRNA‐seq techniques are combined with surface marker expression profiling, known as cellular indexing of transcriptomes and epitopes by sequencing (CITE‐seq) or RNA expression and protein sequencing (REAP‐seq).^189^, ^190^ In these methods, the cells are first labeled using oligonucleotide‐conjugated antibodies for the target surface markers. The oligonucleotides include antibody‐specific barcodes with poly‐A tails to allow their capture using polydT primers in the reverse transcription reaction of scRNA‐seq library construction. Thus, the surface marker expression profile can be measured by counting antibody‐derived barcode sequences. Furthermore, single‐cell multiomics analyses have been developed, such as the simultaneous analysis of transcriptome and chromatin accessibility.^191^ These cutting‐edge analyses have provided valuable resources and important insights into spermatogenesis.

A drawback of scRNA‐seq is that it does not provide spatial information. Recently, spatial transcriptome analysis, which analyzes the transcriptome of the tiny regions of a tissue section following immunohistochemistry or fluorescence in situ hybridization, has made it possible to simultaneously analyze subcellular to single‐cell level transcriptome and spatial information.^192^ This in situ capture‐based approach uses a glass slide that is densely printed with small probe spots. Because probes in each spot have a unique barcode with poly dT sequence, the resulting cDNA with the barcode simultaneously provides transcriptome data with spatial information by placing a tissue section on a glass slide followed by cell permeabilization and reverse transcription. Although reports of spatial transcriptome analysis for spermatogenesis are still few,^193^ the number of the study must increase shortly, providing essential insights.

Single‐cell transcriptome analyses targeting spermatogenesis have provided valuable data and novel insights. As single‐cell technologies and their analysis algorithms are still developing, they are expected to contribute to understanding spermatogenesis.

## CONFLICT OF INTEREST

Takahiro Suzuki declares that he has no conflict of interest.

## HUMAN/ANIMAL RIGHTS STATEMENT AND INFORMATION

This article does not contain any studies with human and animal subjects performed by the author.

## References

[1] Wilhelm D , Yang JX , Thomas P . Mammalian sex determination and gonad development. Curr Top Dev Biol. 2013;106:89–121. 24290348 10.1016/B978-0-12-416021-7.00003-1

[2] Richardson BE , Lehmann R . Mechanisms guiding primordial germ cell migration: strategies from different organisms. Nat Rev Mol Cell Biol. 2010;11:37–49. 20027186 10.1038/nrm2815 PMC4521894

[3] Koopman P , Gubbay J , Vivian N , Goodfellow P , Lovell‐Badge R . Male development of chromosomally female mice transgenic for Sry. Nature. 1991;351:117–21. 2030730 10.1038/351117a0

[4] Sinclair AH , Berta P , Palmer MS , Hawkins JR , Griffiths BL , Smith MJ , et al. A gene from the human sex‐determining region encodes a protein with homology to a conserved DNA‐binding motif. Nature. 1990;346:240–4. 1695712 10.1038/346240a0

[5] Hanley NA , Hagan DM , Clement‐Jones M , Ball SG , Strachan T , Salas‐Cortés L , et al. SRY, SOX9, and DAX1 expression patterns during human sex determination and gonadal development. Mech Dev. 2000;91:403–7. 10704874 10.1016/s0925-4773(99)00307-x

[6] Hacker A , Capel B , Goodfellow P , Lovell‐Badge R . Expression of Sry, the mouse sex determining gene. Development. 1995;121:1603–14. 7600978 10.1242/dev.121.6.1603

[7] Koopman P , Münsterberg A , Capel B , Vivian N , Lovell‐Badge R . Expression of a candidate sex‐determining gene during mouse testis differentiation. Nature. 1990;348:450–2. 2247150 10.1038/348450a0

[8] Jeske YWA , Bowles J , Greenfield A , Koopman P . Expression of a linear Sry transcript in the mouse genital ridge. Nat Genet. 1995;10:480–2. 7670499 10.1038/ng0895-480

[9] McCarrey JR . Toward a more precise and informative nomenclature describing fetal and neonatal male germ cells in rodents. Biol Reprod. 2013;89:47–8. 23843236 10.1095/biolreprod.113.110502 PMC4076367

[10] Yoshida S , Sukeno M , Nakagawa T , Ohbo K , Nagamatsu G , Suda T , et al. The first round of mouse spermatogenesis is a distinctive program that lacks the self‐renewing spermatogonia stage. Development. 2006;133:1495–505. 16540512 10.1242/dev.02316

[11] Fayomi AP , Orwig KE . Spermatogonial stem cells and spermatogenesis in mice, monkeys and men. Stem Cell Res. 2018;29:207–14. 29730571 10.1016/j.scr.2018.04.009 PMC6010318

[12] Phillips BT , Gassei K , Orwig KE . Spermatogonial stem cell regulation and spermatogenesis. Philos Trans R Soc B: Biol Sci. 2010;365:1663–78. 10.1098/rstb.2010.0026 PMC2871929 20403877

[13] Griswold MD . Spermatogenesis: the commitment to meiosis. Physiol Rev. 2016;96:1–17. 26537427 10.1152/physrev.00013.2015 PMC4698398

[14] de Kretser DM , Loveland KL , Meinhardt A , Simorangkir D , Wreford N . Spermatogenesis. Hum Reprod. 1998;13:1–8. 10.1093/humrep/13.suppl_1.1 9663765

[15] Moens PB , Hugenholtz AD . The arrangement of germ cells in the rat seminiferous tubule: an electron‐microscope study. J Cell Sci. 1975;19:487–507. 1206045 10.1242/jcs.19.3.487

[16] Fawcett DW , Ito S , Slautterback D . The occurrence of intercellular bridges in groups of cells exhibiting synchronous differentiation. J Biophys Biochem Cytol. 1959;5:453–60. 13664686 10.1083/jcb.5.3.453 PMC2224676

[17] Huckins C , Oakberg EF . Morphological and quantitative analysis of spermatogonia in mouse testes using whole mounted seminiferous tubules, I. The normal testes. Anat Rec. 1978;192:519–27. 736272 10.1002/ar.1091920406

[18] Basciani S , de Luca G , Dolci S , Brama M , Arizzi M , Mariani S , et al. Platelet‐derived growth factor receptor beta‐subtype regulates proliferation and migration of gonocytes. Endocrinology. 2008;149:6226–35. 18687785 10.1210/en.2008-0349

[19] Orth JM , Qiu J , Jester WF , Pilder S . Expression of the c‐kit gene is critical for migration of neonatal rat gonocytes in vitro. Biol Reprod. 1997;57:676–83. 9283007 10.1095/biolreprod57.3.676

[20] Yang Q‐E , Oatley JM . Early postnatal interactions between Sertoli and germ cells. In: Sertoli Cell Biology. Elsevier; 2015;81–98.

[21] da Ni F , Hao SL , Yang WX . Multiple signaling pathways in Sertoli cells: recent findings in spermatogenesis. Cell Death Dis. 2019;10:1–15. 10.1038/s41419-019-1782-z PMC6637205 31316051

[22] Smith LB , Walker WH . The regulation of spermatogenesis by androgens. Semin Cell Dev Biol. 2014;30:2–13. 24598768 10.1016/j.semcdb.2014.02.012 PMC4043871

[23] Huang Y‐H , Chin C‐C , Ho H‐N , Chou C‐K , Shen C‐N , Kuo H‐C , et al. Pluripotency of mouse spermatogonial stem cells maintained by IGF‐1‐dependent pathway. FASEB J. 2009;23:2076–87. 19246485 10.1096/fj.08-121939

[24] Wang S , Wang X , Wu Y , Han C . IGF‐1R signaling is essential for the proliferation of cultured mouse Spermatogonial stem cells by promoting the G2/M progression of the cell cycle. Stem Cells Dev. 2014;24:471–83. 25356638 10.1089/scd.2014.0376 PMC4313416

[25] Kubota H , Avarbock MR , Brinster RL . Growth factors essential for self‐renewal and expansion of mouse spermatogonial stem cells. Proc Natl Acad Sci USA. 2004;101:16489–94. 15520394 10.1073/pnas.0407063101 PMC534530

[26] Oatley JM , Oatley MJ , Avarbock MR , Tobias JW , Brinster RL . Colony stimulating factor 1 is an extrinsic stimulator of mouse spermatogonial stem cell self‐renewal. Development. 2009;136:1191–9. 19270176 10.1242/dev.032243 PMC2685936

[27] Chen LY , Willis WD , Eddy EM . Targeting the Gdnf gene in peritubular myoid cells disrupts undifferentiated spermatogonial cell development. Proc Natl Acad Sci USA. 2016;113:1829–34. 26831079 10.1073/pnas.1517994113 PMC4763785

[28] Spinnler K , Köhn FM , Schwarzer U , Mayerhofer A . Glial cell line‐derived neurotrophic factor is constitutively produced by human testicular peritubular cells and may contribute to the spermatogonial stem cell niche in man. Hum Reprod. 2010;25:2181–7. 20601681 10.1093/humrep/deq170

[29] Chen LY , Brown PR , Willis WB , Eddy EM . Peritubular Myoid cells participate in male mouse Spermatogonial stem cell maintenance. Endocrinology. 2014;155:4964–74. 25181385 10.1210/en.2014-1406 PMC4239431

[30] DeFalco T , Potter SJ , Williams A v , Waller B , Kan MJ , Capel B . Macrophages contribute to the spermatogonial niche in the adult testis. Cell Rep. 2015;12:1107–19. 26257171 10.1016/j.celrep.2015.07.015 PMC4545310

[31] Lokka E , Lintukorpi L , Cisneros‐Montalvo S , Mäkelä JA , Tyystjärvi S , Ojasalo V , et al. Generation, localization and functions of macrophages during the development of testis. Nat Commun. 2020;11:4375. 32873797 10.1038/s41467-020-18206-0 PMC7463013

[32] Hume DA , Halpin D , Charlton H , Gordon S . The mononuclear phagocyte system of the mouse defined by immunohistochemical localization of antigen F4/80: macrophages of endocrine organs. Proc Natl Acad Sci. 1984;81:4174–7. 6377311 10.1073/pnas.81.13.4174 PMC345391

[33] Hutson JC . Physiologic interactions between macrophages and Leydig cells. Exp Biol Med. 2006;231:1–7. 10.1177/153537020623100101 16380639

[34] Gaytan F , Bellido C , Aguilar E , van Rooijen N . Requirement for testicular macrophages in Leydig cell proliferation and differentiation during prepubertal development in rats. J Reprod Fertil. 1994;102:393–9. 7861393 10.1530/jrf.0.1020393

[35] Cohen PE , Hardy MP , Pollard JW . Colony‐stimulating Factor‐1 plays a major role in the development of reproductive function in male mice. Mol Endocrinol. 1997;11:1636–50. 9328346 10.1210/mend.11.11.0009

[36] Shinohara T , Orwig KE , Avarbock MR , Brinster RL . Remodeling of the postnatal mouse testis is accompanied by dramatic changes in stem cell number and niche accessibility. Proc Natl Acad Sci USA. 2001;98:6186–91. 11371640 10.1073/pnas.111158198 PMC33443

[37] Ota T , Suzuki Y , Nishikawa T , Otsuki T , Sugiyama T , Irie R , et al. Complete sequencing and characterization of 21,243 full‐length human cDNAs. Nat Genet. 2003;36:40–5. 14702039 10.1038/ng1285

[38] Okazaki Y , Furuno M , Kasukawa T , Adachi J , Bono H , Kondo S , et al. Analysis of the mouse transcriptome based on functional annotation of 60,770 full‐length cDNAs. Nature. 2002;420:563–73. 12466851 10.1038/nature01266

[39] Waterston RH , Lindblad‐Toh K , Birney E , Rogers J , Abril JF , Agarwal P , et al. Initial sequencing and comparative analysis of the mouse genome. Nature. 2002;420:520–62. 12466850 10.1038/nature01262

[40] Abdellah Z , Ahmadi A , Ahmed S , Aimable M , Ainscough R , Almeida J , et al. Finishing the euchromatic sequence of the human genome. Nature. 2004;431:931–45. 15496913 10.1038/nature03001

[41] Lander ES , Linton LM , Birren B , Nusbaum C , Zody MC , Baldwin J , et al. Initial sequencing and analysis of the human genome. Nature. 2001;409:860–921. 11237011 10.1038/35057062

[42] Adams MD , Kelley JM , Gocayne JD , Dubnick MAK , Polymeropoulos MH , Xiao H , et al. Complementary DNA sequencing: expressed sequence tags and human genome project. Science (1979). 1991;252:1651–6. 10.1126/science.2047873 2047873

[43] Velculescu VE , Zhang L , Vogelstein B , Kinzler KW . Serial analysis of gene expression. Science (1979). 1995;270:484–7. 10.1126/science.270.5235.484 7570003

[44] Saha S , Sparks AB , Rago C , Akmaev V , Wang CJ , Vogelstein B , et al. Using the transcriptome to annotate the genome. Nat Biotechnol. 2002;20:508–12. 11981567 10.1038/nbt0502-508

[45] Matsumura H , Reich S , Ito A , Saitoh H , Kamoun S , Winter P , et al. Gene expression analysis of plant host‐pathogen interactions by SuperSAGE. Proc Natl Acad Sci USA. 2003;100:15718–23. 14676315 10.1073/pnas.2536670100 PMC307634

[46] Shiraki T , Kondo S , Katayama S , Waki K , Kasukawa T , Kawaji H , et al. Cap analysis gene expression for high‐throughput analysis of transcriptional starting point and identification of promoter usage. Proc Natl Acad Sci USA. 2003;100:15776. 14663149 10.1073/pnas.2136655100 PMC307644

[47] Nguyen QH , Pervolarakis N , Nee K , Kessenbrock K . Experimental considerations for single‐cell RNA sequencing approaches. Front Cell Dev Biol. 2018;6:108. 30234113 10.3389/fcell.2018.00108 PMC6131190

[48] Denisenko E , Guo BB , Jones M , Hou R , de Kock L , Lassmann T , et al. Systematic assessment of tissue dissociation and storage biases in single‐cell and single‐nucleus RNA‐seq workflows. Genome Biol. 2020;21:1–25. 10.1186/s13059-020-02048-6 PMC7265231 32487174

[49] Adam M , Potter AS , Potter SS . Psychrophilic proteases dramatically reduce single‐cell RNA‐seq artifacts: a molecular atlas of kidney development. Development. 2017;144:3625–32. 28851704 10.1242/dev.151142 PMC5665481

[50] Saliba AE , Westermann AJ , Gorski SA , Vogel J . Single‐cell RNA‐seq: advances and future challenges. Nucleic Acids Res. 2014;42:8845–60. 25053837 10.1093/nar/gku555 PMC4132710

[51] Mora‐Castilla S , To C , Vaezeslami S , Morey R , Srinivasan S , Dumdie JN , et al. Miniaturization technologies for efficient single‐cell library preparation for next‐generation sequencing. J Lab Autom. 2016;21:557–67. 26891732 10.1177/2211068216630741 PMC4948133

[52] Foley JW , Zhu C , Jolivet P , Zhu SX , Lu P , Meaney MJ , et al. Gene expression profiling of single cells from archival tissue with laser‐capture microdissection and Smart‐3SEQ. Genome Res. 2019;29:1816–25. 31519740 10.1101/gr.234807.118 PMC6836736

[53] Zheng GXY , Terry JM , Belgrader P , Ryvkin P , Bent ZW , Wilson R , et al. Massively parallel digital transcriptional profiling of single cells. Nat Commun. 2017;8:1–12. 28091601 10.1038/ncomms14049 PMC5241818

[54] Rosenberg AB , Roco CM , Muscat RA , Kuchina A , Sample P , Yao Z , et al. Single‐cell profiling of the developing mouse brain and spinal cord with split‐pool barcoding. Science (1979). 2018;360:176–82. 10.1126/science.aam8999 PMC7643870 29545511

[55] Ramsköld D , Luo S , Wang YC , Li R , Deng Q , Faridani OR , et al. Full‐length mRNA‐Seq from single‐cell levels of RNA and individual circulating tumor cells. Nat Biotechnol. 2012;30:777–82. 22820318 10.1038/nbt.2282 PMC3467340

[56] Hagemann‐Jensen M , Ziegenhain C , Chen P , Ramsköld D , Hendriks GJ , Larsson AJM , et al. Single‐cell RNA counting at allele and isoform resolution using Smart‐seq3. Nat Biotechnol. 2020;38:708–14. 32518404 10.1038/s41587-020-0497-0

[57] Picelli S , Björklund ÅK , Faridani OR , Sagasser S , Winberg G , Sandberg R . Smart‐seq2 for sensitive full‐length transcriptome profiling in single cells. Nat Methods. 2013;10:1096–8. 24056875 10.1038/nmeth.2639

[58] Picelli S , Faridani OR , Björklund ÅK , Winberg G , Sagasser S , Sandberg R . Full‐length RNA‐seq from single cells using Smart‐seq2. Nat Protoc. 2014;9:171–81. 24385147 10.1038/nprot.2014.006

[59] Hahaut V , Pavlinic D , Carbone W , Schuierer S , Balmer P , Quinodoz M , et al. Fast and highly sensitive full‐length single‐cell RNA sequencing using FLASH‐seq. Nat Biotechnol. 2022;40:1447–51. 35637419 10.1038/s41587-022-01312-3 PMC9546769

[60] Sibley CR , Blazquez L , Ule J . Lessons from non‐canonical splicing. Nat Rev Genet. 2016;17:407–21. 27240813 10.1038/nrg.2016.46 PMC5154377

[61] Hayashi T , Ozaki H , Sasagawa Y , Umeda M , Danno H , Nikaido I . Single‐cell full‐length total RNA sequencing uncovers dynamics of recursive splicing and enhancer RNAs. Nat Commun. 2018;9:1–16. 29434199 10.1038/s41467-018-02866-0 PMC5809388

[62] Jaitin DA , Kenigsberg E , Keren‐Shaul H , Elefant N , Paul F , Zaretsky I , et al. Massively parallel single‐cell RNA‐seq for marker‐free decomposition of tissues into cell types. Science (1979). 2014;343:776–9. 10.1126/science.1247651 PMC4412462 24531970

[63] Macosko EZ , Basu A , Satija R , Nemesh J , Shekhar K , Goldman M , et al. Highly parallel genome‐wide expression profiling of individual cells using nanoliter droplets. Cell. 2015;161:1202–14. 26000488 10.1016/j.cell.2015.05.002 PMC4481139

[64] Klein AM , Mazutis L , Akartuna I , Tallapragada N , Veres A , Li V , et al. Droplet barcoding for single‐cell transcriptomics applied to embryonic stem cells. Cell. 2015;161:1187–201. 26000487 10.1016/j.cell.2015.04.044 PMC4441768

[65] Hashimshony T , Senderovich N , Avital G , Klochendler A , de Leeuw Y , Anavy L , et al. CEL‐Seq2: sensitive highly‐multiplexed single‐cell RNA‐Seq. Genome Biol. 2016;17:1–7. 27121950 10.1186/s13059-016-0938-8 PMC4848782

[66] Bagnoli JW , Ziegenhain C , Janjic A , Wange LE , Vieth B , Parekh S , et al. Sensitive and powerful single‐cell RNA sequencing using mcSCRB‐seq. Nat Commun. 2018;9:1–8. 30050112 10.1038/s41467-018-05347-6 PMC6062574

[67] Sasagawa Y , Danno H , Takada H , Ebisawa M , Tanaka K , Hayashi T , et al. Quartz‐Seq2: a high‐throughput single‐cell RNA‐sequencing method that effectively uses limited sequence reads. Genome Biol. 2018;19:1–24. 29523163 10.1186/s13059-018-1407-3 PMC5845169

[68] Kivioja T , Vähärautio A , Karlsson K , Bonke M , Enge M , Linnarsson S , et al. Counting absolute numbers of molecules using unique molecular identifiers. Nat Methods. 2011;9:72–4. 22101854 10.1038/nmeth.1778

[69] Islam S , Zeisel A , Joost S , la Manno G , Zajac P , Kasper M , et al. Quantitative single‐cell RNA‐seq with unique molecular identifiers. Nat Methods. 2014;11:163–6. 24363023 10.1038/nmeth.2772

[70] Wang X , He Y , Zhang Q , Ren X , Zhang Z . Direct comparative analyses of 10X genomics chromium and Smart‐seq2. Genomics Proteomics Bioinformatics. 2021;19:253–66. 33662621 10.1016/j.gpb.2020.02.005 PMC8602399

[71] Mereu E , Lafzi A , Moutinho C , Ziegenhain C , McCarthy DJ , Álvarez‐Varela A , et al. Benchmarking single‐cell RNA‐sequencing protocols for cell atlas projects. Nat Biotechnol. 2020;38:747–55. 32518403 10.1038/s41587-020-0469-4

[72] Ziegenhain C , Vieth B , Parekh S , Reinius B , Guillaumet‐Adkins A , Smets M , et al. Comparative analysis of single‐cell RNA sequencing methods. Mol Cell. 2017;65:631–643.e4. 28212749 10.1016/j.molcel.2017.01.023

[73] Hao Y , Hao S , Andersen‐Nissen E , Mauck WM , Zheng S , Butler A , et al. Integrated analysis of multimodal single‐cell data. Cell. 2021;184:3573–3587.e29. 34062119 10.1016/j.cell.2021.04.048 PMC8238499

[74] Stuart T , Butler A , Hoffman P , Hafemeister C , Papalexi E , Mauck WM , et al. Comprehensive integration of single‐cell data. Cell. 2019;177:1888–1902.e21. 31178118 10.1016/j.cell.2019.05.031 PMC6687398

[75] Butler A , Hoffman P , Smibert P , Papalexi E , Satija R . Integrating single‐cell transcriptomic data across different conditions, technologies, and species. Nat Biotechnol. 2018;36:411–20. 29608179 10.1038/nbt.4096 PMC6700744

[76] Satija R , Farrell JA , Gennert D , Schier AF , Regev A . Spatial reconstruction of single‐cell gene expression data. Nat Biotechnol. 2015;33:495–502. 25867923 10.1038/nbt.3192 PMC4430369

[77] Kiselev VY , Kirschner K , Schaub MT , Andrews T , Yiu A , Chandra T , et al. SC3: consensus clustering of single‐cell RNA‐seq data. Nat Methods. 2017;14:483–6. 28346451 10.1038/nmeth.4236 PMC5410170

[78] Wolf FA , Angerer P , Theis FJ . SCANPY: Large‐scale single‐cell gene expression data analysis. Genome Biol. 2018;19:1–5. 29409532 10.1186/s13059-017-1382-0 PMC5802054

[79] Coifman RR , Lafon S , Lee AB , Maggioni M , Nadler B , Warner F , et al. Geometric diffusions as a tool for harmonic analysis and structure definition of data: diffusion maps. Proc Natl Acad Sci USA. 2005;102:7426–31. 15899970 10.1073/pnas.0500334102 PMC1140422

[80] van der Maaten L , Hinton G . Visualizing Data using t‐SNE. J Mach Learn Res. 2008;9:2579–605.

[81] McInnes L , Healy J , Saul N , Großberger L . UMAP: uniform manifold approximation and projection. J Open Source Softw. 2018;3:861.

[82] Becht E , McInnes L , Healy J , Dutertre CA , Kwok IWH , Ng LG , et al. Dimensionality reduction for visualizing single‐cell data using UMAP. Nat Biotechnol. 2018;37:38–44. 10.1038/nbt.4314 30531897

[83] Qiu X , Mao Q , Tang Y , Wang L , Chawla R , Pliner HA , et al. Reversed graph embedding resolves complex single‐cell trajectories. Nat Methods. 2017;14(10):979–82. 28825705 10.1038/nmeth.4402 PMC5764547

[84] Grün D , Lyubimova A , Kester L , Wiebrands K , Basak O , Sasaki N , et al. Single‐cell messenger RNA sequencing reveals rare intestinal cell types. Nature. 2015;525:251–5. 26287467 10.1038/nature14966

[85] Lin P , Troup M , Ho JWK . CIDR: ultrafast and accurate clustering through imputation for single‐cell RNA‐seq data. Genome Biol. 2017;18:1–11. 28351406 10.1186/s13059-017-1188-0 PMC5371246

[86] Chen J , Schlitzer A , Chakarov S , Ginhoux F , Poidinger M . Mpath maps multi‐branching single‐cell trajectories revealing progenitor cell progression during development. Nat Commun. 2016;7:1–15. 10.1038/ncomms11988 PMC4931327 27356503

[87] Kiselev VY , Andrews TS , Hemberg M . Challenges in unsupervised clustering of single‐cell RNA‐seq data. Nat Rev Genet. 2019;20:273–82. 30617341 10.1038/s41576-018-0088-9

[88] Hou R , Denisenko E , Forrest ARR . scMatch: a single‐cell gene expression profile annotation tool using reference datasets. Bioinformatics. 2019;35:4688–95. 31028376 10.1093/bioinformatics/btz292 PMC6853649

[89] Yuan M , Chen L , Deng M . scMRA: a robust deep learning method to annotate scRNA‐seq data with multiple reference datasets. Bioinformatics. 2022;38:738–45. 10.1093/bioinformatics/btab700 34623390

[90] Ma F , Pellegrini M . ACTINN: automated identification of cell types in single cell RNA sequencing. Bioinformatics. 2020;36:533–8. 31359028 10.1093/bioinformatics/btz592

[91] Shao X , Yang H , Zhuang X , Liao J , Yang P , Cheng J , et al. scDeepSort: a pre‐trained cell‐type annotation method for single‐cell transcriptomics using deep learning with a weighted graph neural network. Nucleic Acids Res. 2021;49:e122–2. 34500471 10.1093/nar/gkab775 PMC8643674

[92] Squair JW , Gautier M , Kathe C , Anderson MA , James ND , Hutson TH , et al. Confronting false discoveries in single‐cell differential expression. Nat Commun. 2021;12:1–15. 34584091 10.1038/s41467-021-25960-2 PMC8479118

[93] Arzalluz‐Luque Á , Devailly G , Mantsoki A , Joshi A . Delineating biological and technical variance in single cell expression data. Int J Biochem Cell Biol. 2017;90:161. 28716546 10.1016/j.biocel.2017.07.006 PMC5608017

[94] Ji Z , Ji H . TSCAN: pseudo‐time reconstruction and evaluation in single‐cell RNA‐seq analysis. Nucleic Acids Res. 2016;44:e117–7. 27179027 10.1093/nar/gkw430 PMC4994863

[95] Street K , Risso D , Fletcher RB , Das D , Ngai J , Yosef N , et al. Slingshot: cell lineage and pseudotime inference for single‐cell transcriptomics. BMC Genomics. 2018;19:1–16. 29914354 10.1186/s12864-018-4772-0 PMC6007078

[96] Campbell KR , Yau C . Uncovering pseudotemporal trajectories with covariates from single cell and bulk expression data. Nat Commun. 2018;9:1–12. 29934517 10.1038/s41467-018-04696-6 PMC6015076

[97] van den Berge K , Roux de Bézieux H , Street K , Saelens W , Cannoodt R , Saeys Y , et al. Trajectory‐based differential expression analysis for single‐cell sequencing data. Nat Commun. 2020;11:1–13. 32139671 10.1038/s41467-020-14766-3 PMC7058077

[98] Saelens W , Cannoodt R , Todorov H , Saeys Y . A comparison of single‐cell trajectory inference methods. Nat Biotechnol. 2019;37:547–54. 30936559 10.1038/s41587-019-0071-9

[99] Cao J , Spielmann M , Qiu X , Huang X , Ibrahim DM , Hill AJ , et al. The single‐cell transcriptional landscape of mammalian organogenesis. Nature. 2019;566:496–502. 30787437 10.1038/s41586-019-0969-x PMC6434952

[100] Trapnell C , Cacchiarelli D , Grimsby J , Pokharel P , Li S , Morse M , et al. The dynamics and regulators of cell fate decisions are revealed by pseudotemporal ordering of single cells. Nat Biotechnol. 2014;32:381–6. 24658644 10.1038/nbt.2859 PMC4122333

[101] Wolf FA , Hamey FK , Plass M , Solana J , Dahlin JS , Göttgens B , et al. PAGA: graph abstraction reconciles clustering with trajectory inference through a topology preserving map of single cells. Genome Biol. 2019;20:1–9. 30890159 10.1186/s13059-019-1663-x PMC6425583

[102] la Manno G , Soldatov R , Zeisel A , Braun E , Hochgerner H , Petukhov V , et al. RNA velocity of single cells. Nature. 2018;560:494–8. 30089906 10.1038/s41586-018-0414-6 PMC6130801

[103] Bergen V , Lange M , Peidli S , Wolf FA , Theis FJ . Generalizing RNA velocity to transient cell states through dynamical modeling. Nat Biotechnol. 2020;38:1408–14. 32747759 10.1038/s41587-020-0591-3

[104] Green CD , Ma Q , Manske GL , Shami AN , Zheng X , Marini S , et al. A comprehensive roadmap of murine spermatogenesis defined by single‐cell RNA‐Seq. Dev Cell. 2018;46:651–667.e10. 30146481 10.1016/j.devcel.2018.07.025 PMC6713459

[105] Chi SY , Shami AN , Moritz L , Larose H , Manske GL , Ma Q , et al. TCF21+ mesenchymal cells contribute to testis somatic cell development, homeostasis, and regeneration in mice. Nat Commun. 2021;12:1–17. 34162856 10.1038/s41467-021-24130-8 PMC8222243

[106] Chen P , Zhao X , Guan X , Chen H . Origin and regulation of stem Leydig cells in the adult testis. Curr Opin Endocr Metab Res. 2019;6:49–53.

[107] Cool J , Carmona FD , Szucsik JC , Capel B . Peritubular Myoid cells are not the migrating population required for testis cord formation in the XY gonad. Sex Dev. 2008;2:128–33. 18769072 10.1159/000143430 PMC2683975

[108] Guo J , Nie X , Giebler M , Mlcochova H , Wang Y , Grow EJ , et al. The dynamic transcriptional cell atlas of testis development during human puberty. Cell Stem Cell. 2020;26:262–276.e4. 31928944 10.1016/j.stem.2019.12.005 PMC7298616

[109] Tan K , Song HW , Wilkinson MF . Single‐cell RNAseq analysis of testicular germ and somatic cell development during the perinatal period. Development. 2020;147:dev183251. 31964773 10.1242/dev.183251 PMC7033731

[110] Kobayashi T , Motomura Y , Moro K . The discovery of group 2 innate lymphoid cells has changed the concept of type 2 immune diseases. Int Immunol. 2021;33:705–9. 34498700 10.1093/intimm/dxab063 PMC8633664

[111] Sohni A , Tan K , Song HW , Burow D , de Rooij DG , Laurent L , et al. The neonatal and adult human testis defined at the single‐cell level. Cell Rep. 2019;26:1501–1517.e4. 30726734 10.1016/j.celrep.2019.01.045 PMC6402825

[112] Guo J , Grow EJ , Mlcochova H , Maher GJ , Lindskog C , Nie X , et al. The adult human testis transcriptional cell atlas. Cell Res. 2018;28:1141–57. 30315278 10.1038/s41422-018-0099-2 PMC6274646

[113] Hermann BP , Cheng K , Singh A , Roa‐De La Cruz L , Mutoji KN , Chen IC , et al. The mammalian spermatogenesis single‐cell transcriptome, from Spermatogonial stem cells to spermatids. Cell Rep. 2018;25:1650–1667.e8. 30404016 10.1016/j.celrep.2018.10.026 PMC6384825

[114] Wang M , Liu X , Chang G , Chen Y , An G , Yan L , et al. Single‐cell RNA sequencing analysis reveals sequential cell fate transition during human spermatogenesis. Cell Stem Cell. 2018;23:599–614.e4. 30174296 10.1016/j.stem.2018.08.007

[115] Guo J , Grow EJ , Yi C , Mlcochova H , Maher GJ , Lindskog C , et al. Chromatin and single‐cell RNA‐Seq profiling reveal dynamic signaling and metabolic transitions during human Spermatogonial stem cell development. Cell Stem Cell. 2017;21:533–546.e6. 28985528 10.1016/j.stem.2017.09.003 PMC5832720

[116] Li L , Dong J , Yan L , Yong J , Liu X , Hu Y , et al. Single‐cell RNA‐Seq analysis maps development of human germline cells and gonadal niche interactions. Cell Stem Cell. 2017;20:858–873.e4. 28457750 10.1016/j.stem.2017.03.007

[117] Zhao J , Lu P , Wan C , Huang Y , Cui M , Yang X , et al. Cell‐fate transition and determination analysis of mouse male germ cells throughout development. Nat Commun. 2021;12:1–20. 34824237 10.1038/s41467-021-27172-0 PMC8617176

[118] Grive KJ , Hu Y , Shu E , Grimson A , Elemento O , Grenier JK , et al. Dynamic transcriptome profiles within spermatogonial and spermatocyte populations during postnatal testis maturation revealed by single‐cell sequencing. PLoS Genet. 2019;15:e1007810. 30893341 10.1371/journal.pgen.1007810 PMC6443194

[119] Chen Y , Zheng Y , Gao Y , Lin Z , Yang S , Wang T , et al. Single‐cell RNA‐seq uncovers dynamic processes and critical regulators in mouse spermatogenesis. Cell Res. 2018;28:879–96. 30061742 10.1038/s41422-018-0074-y PMC6123400

[120] Lukassen S , Bosch E , Ekici AB , Winterpacht A . Characterization of germ cell differentiation in the male mouse through single‐cell RNA sequencing. Sci Rep. 2018;8:1–7. 29695820 10.1038/s41598-018-24725-0 PMC5916943

[121] Guo J , Sosa E , Chitiashvili T , Nie X , Rojas EJ , Oliver E , et al. Single‐cell analysis of the developing human testis reveals somatic niche cell specification and fetal germline stem cell establishment. Cell Stem Cell. 2021;28:764–778.e4. 33453151 10.1016/j.stem.2020.12.004 PMC8026516

[122] Guo F , Yan L , Guo H , Li L , Hu B , Zhao Y , et al. The transcriptome and DNA Methylome landscapes of human primordial germ cells. Cell. 2015;161:1437–52. 26046443 10.1016/j.cell.2015.05.015

[123] Kadam P , Ntemou E , Onofre J , van Saen D , Goossens E . Does co‐transplantation of mesenchymal and spermatogonial stem cells improve reproductive efficiency and safety in mice? Stem Cell Res Ther. 2019;10:1–10. 31640769 10.1186/s13287-019-1420-9 PMC6805426

[124] Shinohara T , Ishii K , Kanatsu‐Shinohara M . Unstable side population phenotype of mouse spermatogonial stem cells in vitro. J Reprod Dev. 2011;57:288–95. 21224526 10.1262/jrd.10-168n

[125] Morimoto H , Kanatsu‐Shinohara M , Takashima S , Chuma S , Nakatsuji N , Takehashi M , et al. Phenotypic plasticity of mouse Spermatogonial stem cells. PLoS One. 2009;4:e7909. 19936070 10.1371/journal.pone.0007909 PMC2774941

[126] Yoshida S , Nabeshima YI , Nakagawa T . Stem Cell Heterogeneity. Ann N Y Acad Sci. 2007;1120:47–58. 17905929 10.1196/annals.1411.003

[127] Oatley MJ , Kaucher A v , Racicot KE , Oatley JM . Inhibitor of DNA binding 4 is expressed selectively by single spermatogonia in the male germline and regulates the self‐renewal of spermatogonial stem cells in mice. Biol Reprod. 2011;85:347–56. 21543770 10.1095/biolreprod.111.091330 PMC3142260

[128] Nakagawa T , Sharma M , Nabeshima YI , Braun RE , Yoshida S . Functional hierarchy and reversibility within the murine spermatogenic stem cell compartment. Science (1979). 2010;328:62–7. 10.1126/science.1182868 PMC2981100 20299552

[129] Komai Y , Tanaka T , Tokuyama Y , Yanai H , Ohe S , Omachi T , et al. Bmi1 expression in long‐term germ stem cells. Sci Rep. 2014;4:1–12. 10.1038/srep06175 PMC4141270 25146451

[130] Aloisio GM , Nakada Y , Saatcioglu HD , Peña CG , Baker MD , Tarnawa ED , et al. PAX7 expression defines germline stem cells in the adult testis. J Clin Invest. 2014;124:3929–44. 25133429 10.1172/JCI75943 PMC4153705

[131] Chan F , Oatley MJ , Kaucher A v , Yang QE , Bieberich CJ , Shashikant CS , et al. Functional and molecular features of the Id4+ germline stem cell population in mouse testes. Genes Dev. 2014;28:1351–62. 24939937 10.1101/gad.240465.114 PMC4066404

[132] Zheng K , Wu X , Kaestner KH , Wang PJ . The pluripotency factor LIN28 marks undifferentiated spermatogonia in mouse. BMC Dev Biol. 2009;9:1–11. 19563657 10.1186/1471-213X-9-38 PMC2719617

[133] Grisanti L , Falciatori I , Grasso M , Dovere L , Fera S , Muciaccia B , et al. Identification of Spermatogonial stem cell subsets by morphological analysis and prospective isolation. Stem Cells. 2009;27:3043–52. 19711452 10.1002/stem.206

[134] Suzuki H , Sada A , Yoshida S , Saga Y . The heterogeneity of spermatogonia is revealed by their topology and expression of marker proteins including the germ cell‐specific proteins Nanos2 and Nanos3. Dev Biol. 2009;336:222–31. 19818747 10.1016/j.ydbio.2009.10.002

[135] Hermann BP , Mutoji KN , Velte EK , Ko D , Oatley JM , Geyer CB , et al. Transcriptional and translational heterogeneity among neonatal mouse spermatogonia. Biol Reprod. 2015;92:1–12. 10.1095/biolreprod.114.125757 PMC4342790 25568304

[136] Helsel AR , Yang QE , Oatley MJ , Lord T , Sablitzky F , Oatley JM . Id4 levels dictate the stem cell state in mouse spermatogonia. Development. 2017;144:624–34. 28087628 10.1242/dev.146928 PMC5312040

[137] Nakagawa T , Nabeshima Y , Yoshida S . Functional identification of the actual and potential stem cell compartments in mouse spermatogenesis. Dev Cell. 2007;12:195–206. 17276338 10.1016/j.devcel.2007.01.002

[138] Barroca V , Lassalle B , Coureuil M , Louis JP , le Page F , Testart J , et al. Mouse differentiating spermatogonia can generate germinal stem cells in vivo. Nat Cell Biol. 2008;11:190–6. 19098901 10.1038/ncb1826

[139] Yoshida S . Casting back to stem cells. Nat Cell Biol. 2009;11:118–20. 19188918 10.1038/ncb0209-118

[140] Shea KL , Xiang W , LaPorta VS , Licht JD , Keller C , Basson MA , et al. Sprouty1 regulates reversible quiescence of a self‐renewing adult muscle stem cell Pool during regeneration. Cell Stem Cell. 2010;6:117–29. 20144785 10.1016/j.stem.2009.12.015 PMC2846417

[141] Glickstein SB , Monaghan JA , Koeller HB , Jones TK , Ross ME . Cyclin D2 is critical for intermediate progenitor cell proliferation in the embryonic cortex. J Neurosci. 2009;29:9614–24. 19641124 10.1523/JNEUROSCI.2284-09.2009 PMC2811167

[142] Mueller JL , Mahadevaiah SK , Park PJ , Warburton PE , Page DC , Turner JMA . The mouse X chromosome is enriched for multicopy testis genes showing postmeiotic expression. Nat Genet. 2008;40:794–9. 18454149 10.1038/ng.126 PMC2740655

[143] Kierszenbaum AL , Rivkin E , Tres LL , Duan C , Goldberg E , Szot M , et al. Molecular aspects of XY body formation. Cytogenet Genome Res. 2003;103:245–55. 15051945 10.1159/000076810

[144] Handel MA . The XY body: a specialized meiotic chromatin domain. Exp Cell Res. 2004;296:57–63. 15120994 10.1016/j.yexcr.2004.03.008

[145] Wang PJ , Page DC , McCarrey JR . Differential expression of sex‐linked and autosomal germ‐cell‐specific genes during spermatogenesis in the mouse. Hum Mol Genet. 2005;14:2911–8. 16118233 10.1093/hmg/ddi322 PMC1994333

[146] Turner JMA , Mahadevaiah SK , Fernandez‐Capetillo O , Nussenzweig A , Xu X , Deng CX , et al. Silencing of unsynapsed meiotic chromosomes in the mouse. Nat Genet. 2004;37:41–7. 15580272 10.1038/ng1484

[147] Ernst C , Eling N , Martinez‐Jimenez CP , Marioni JC , Odom DT . Staged developmental mapping and X chromosome transcriptional dynamics during mouse spermatogenesis. Nat Commun. 2019;10:1–20. 30890697 10.1038/s41467-019-09182-1 PMC6424977

[148] Turner JMA . Meiotic silencing in mammals. Annu Rev Genet. 2015;49:395–412. 26631513 10.1146/annurev-genet-112414-055145

[149] Namekawa SH , Park PJ , Zhang LF , Shima JE , McCarrey JR , Griswold MD , et al. Postmeiotic sex chromatin in the male germline of mice. Curr Biol. 2006;16:660–7. 16581510 10.1016/j.cub.2006.01.066

[150] Morales CR , Wu XQ , Hecht NB . The DNA/RNA‐binding protein, TB‐RBP, moves from the nucleus to the cytoplasm and through intercellular bridges in male germ cells. Dev Biol. 1998;201:113–23. 9733578 10.1006/dbio.1998.8967

[151] Caldwell KA , Handel MA . Protamine transcript sharing among postmeiotic spermatids. Proc Natl Acad Sci USA. 1991;88:2407–11. 2006178 10.1073/pnas.88.6.2407 PMC51241

[152] Braun RE , Behringer RR , Peschon JJ , Brinster RL , Palmiter RD . Genetically haploid spermatids are phenotypically diploid. Nature. 1989;337:373–6. 2911388 10.1038/337373a0

[153] Nef S , Schaad O , Stallings NR , Cederroth CR , Pitetti JL , Schaer G , et al. Gene expression during sex determination reveals a robust female genetic program at the onset of ovarian development. Dev Biol. 2005;287:361–77. 16214126 10.1016/j.ydbio.2005.09.008

[154] Stévant I , Neirijnck Y , Borel C , Escoffier J , Smith LB , Antonarakis SE , et al. Deciphering cell lineage specification during male sex determination with single‐cell RNA sequencing. Cell Rep. 2018;22:1589–99. 29425512 10.1016/j.celrep.2018.01.043

[155] Hu YC , Okumura LM , Page DC . Gata4 is required for formation of the genital ridge in mice. PLoS Genet. 2013;9:e1003629. 23874227 10.1371/journal.pgen.1003629 PMC3708810

[156] Tevosian SG , Albrecht KH , Crispino JD , Fujiwara Y , Eicher EM , Orkin SH . Gonadal differentiation, sex determination and normal Sry expression in mice require direct interaction between transcription partners GATA4 and FOG2. Development. 2002;129:4627–34. 12223418 10.1242/dev.129.19.4627

[157] Hammes A , Guo JK , Lutsch G , Leheste JR , Landrock D , Ziegler U , et al. Two splice variants of the wilms' tumor 1 gene have distinct functions during sex determination and nephron formation. Cell. 2001;106:319–29. 11509181 10.1016/s0092-8674(01)00453-6

[158] Lin YT , Barske L , Defalco T , Capel B . Numb regulates somatic cell lineage commitment during early gonadogenesis in mice. Development. 2017;144:1607. 28360133 10.1242/dev.149203 PMC5450849

[159] Liu C , Rodriguez K , Yao HHC . Mapping lineage progression of somatic progenitor cells in the mouse fetal testis. Development. 2016;143:3700–10. 27621062 10.1242/dev.135756 PMC5087644

[160] Vergouwen RPFA , Jacobs SGPM , Huiskamp R , Davids JAG , de Rooij DG . Proliferative activity of gonocytes, Sertoli cells and interstitial cells during testicular development in mice. Reproduction. 1991;93:233–43. 10.1530/jrf.0.0930233 1920294

[161] Mayère C , Regard V , Perea‐Gomez A , Bunce C , Neirijnck Y , Djari C , et al. Origin, specification and differentiation of a rare supporting‐like lineage in the developing mouse gonad. Sci Adv. 2022;8:972. 10.1126/sciadv.abm0972 PMC10942771 35613264

[162] Griswold SL , Behringer RR . Fetal Leydig cell origin and development. Sex Dev. 2009;3:1–15. 19339813 10.1159/000200077 PMC4021856

[163] Kerr JB , Knell CM . The fate of fetal Leydig cells during the development of the fetal and postnatal rat testis. Development. 1988;103:535–44. 3246223 10.1242/dev.103.3.535

[164] Ariyaratne HBS , Mendis‐Handagama SMLC . Changes in the testis Interstitium of Sprague Dawley rats from birth to sexual maturity. Biol Reprod. 2000;62:680–90. 10684810 10.1095/biolreprod62.3.680

[165] Mendis‐HanfAagama SMLC , Riabeidger GP , Kretser DMD . Morphometric analysis of the components of the neonatal and the adult rat testis interstitium. Int J Androl. 1987;10:525–34. 3610361 10.1111/j.1365-2605.1987.tb00352.x

[166] Shima Y , Matsuzaki S , Miyabayashi K , Otake H , Baba T , Kato S , et al. Fetal Leydig cells persist as an androgen‐independent subpopulation in the postnatal testis. Mol Endocrinol. 2015;29:1581–93. 26402718 10.1210/me.2015-1200 PMC5414671

[167] Kaftanovskaya EM , Lopez C , Ferguson L , Myhr C , Agoulnik AI . Genetic ablation of androgen receptor signaling in fetal Leydig cell lineage affects Leydig cell functions in adult testis. FASEB J. 2015;29:2327–37. 25713029 10.1096/fj.14-263632 PMC6137449

[168] Oakberg EF . Spermatogonial stem‐cell renewal in the mouse. Anat Rec. 1971;169:515–31. 5550531 10.1002/ar.1091690305

[169] Ahmed EA , de Rooij DG . Staging of mouse seminiferous tubule cross‐sections. Methods Mol Biol. 2009;558:263–77. 19685330 10.1007/978-1-60761-103-5_16

[170] Oakberg EF . Duration of spermatogenesis in the mouse. Nature. 1957;180:1137–8. 13483640 10.1038/1801137a0

[171] Oakberg EF . Duration of spermatogenesis in the mouse and timing of stages of the cycle of the seminiferous epithelium. Am J Anat. 1956;99:507–16. 13402729 10.1002/aja.1000990307

[172] Endo T , Freinkman E , de Rooij DG , Page DC . Periodic production of retinoic acid by meiotic and somatic cells coordinates four transitions in mouse spermatogenesis. Proc Natl Acad Sci USA. 2017;114:E10132–41. 29109271 10.1073/pnas.1710837114 PMC5703301

[173] Kerr JB . An ultrastructural and morphometric analysis of the Sertoli cell during the spermatogenic cycle of the rat. Anat Embryol (Berl). 1988;179:191–203. 3232856 10.1007/BF00304701

[174] Kerr JB . A light microscopic and morphometric analysis of the Sertoli cell during the spermatogenic cycle of the rat. Anat Embryol (Berl). 1988;177:341–8. 3354850 10.1007/BF00315842

[175] Johnston DS , Wright WW , DiCandeloro P , Wilson E , Kopf GS , Jelinsky SA . Stage‐specific gene expression is a fundamental characteristic of rat spermatogenic cells and Sertoli cells. Proc Natl Acad Sci USA. 2008;105:8315–20. 18544648 10.1073/pnas.0709854105 PMC2448834

[176] Hasegawa K , Saga Y . Retinoic acid signaling in Sertoli cells regulates organization of the blood‐testis barrier through cyclical changes in gene expression. Development. 2012;139:4347–55. 23095883 10.1242/dev.080119

[177] Gewiss RL , Law NC , Helsel AR , Shelden EA , Griswold MD . Two distinct Sertoli cell states are regulated via germ cell crosstalk. Biol Reprod. 2021;105:1591–602. 34494084 10.1093/biolre/ioab160 PMC8689118

[178] Tsuyuzaki K , Ishii M , Nikaido I . Uncovering hypergraphs of cell–cell interaction from single cell RNA‐sequencing data. *bioRxiv* . 2019; 10.1101/566182. PMC10631077 37936079

[179] Efremova M , Vento‐Tormo M , Teichmann SA , Vento‐Tormo R . CellPhoneDB: inferring cell–cell communication from combined expression of multi‐subunit ligand–receptor complexes. Nat Protoc. 2020;15:1484–506. 32103204 10.1038/s41596-020-0292-x

[180] Jin S , Guerrero‐Juarez CF , Zhang L , Chang I , Ramos R , Kuan CH , et al. Inference and analysis of cell‐cell communication using CellChat. Nat Commun. 2021;12:1–20. 33597522 10.1038/s41467-021-21246-9 PMC7889871

[181] Garcia TX , Hofmann MC . NOTCH signaling in Sertoli cells regulates gonocyte fate. Cell Cycle. 2013;12:2538. 23907117 10.4161/cc.25627 PMC3865042

[182] Garcia TX , DeFalco T , Capel B , Hofmann MC . Constitutive activation of NOTCH1 signaling in Sertoli cells causes gonocyte exit from quiescence. Dev Biol. 2013;377:188–201. 23391689 10.1016/j.ydbio.2013.01.031 PMC3630254

[183] Nicholls PK , Stanton PG , Chen JL , Olcorn JS , Haverfield JT , Qian H , et al. Activin signaling regulates Sertoli cell differentiation and function. Endocrinology. 2012;153:6065–77. 23117933 10.1210/en.2012-1821

[184] Buzzard JJ , Farnworth PG , de Kretser DM , O'Connor AE , Wreford NG , Morrison JR . Proliferative phase Sertoli cells display a developmentally regulated response to Activin in vitro. Endocrinology. 2003;144:474–83. 12538607 10.1210/en.2002-220595

[185] Mendis SHS , Meachem SJ , Sarraj MA , Loveland KL . Activin a balances Sertoli and germ cell proliferation in the fetal mouse testis. Biol Reprod. 2011;84:379–91. 20926807 10.1095/biolreprod.110.086231

[186] Wijayarathna R , de Kretser DM . Activins in reproductive biology and beyond. Hum Reprod Update. 2016;22:342–57. 26884470 10.1093/humupd/dmv058

[187] Radicioni AF , Anzuini A , de Marco E , Nofroni I , Castracane VD , Lenzi A . Changes in serum inhibin B during normal male puberty. Eur J Endocrinol. 2005;152:403–9. 15757857 10.1530/eje.1.01855

[188] Zhao LY , Yao CC , Xing XY , Jing T , Li P , Zhu ZJ , et al. Single‐cell analysis of developing and azoospermia human testicles reveals central role of Sertoli cells. Nat Commun. 2020;11:1–15. 33173058 10.1038/s41467-020-19414-4 PMC7655944

[189] Stoeckius M , Hafemeister C , Stephenson W , Houck‐Loomis B , Chattopadhyay PK , Swerdlow H , et al. Simultaneous epitope and transcriptome measurement in single cells. Nat Methods. 2017;14:865–8. 28759029 10.1038/nmeth.4380 PMC5669064

[190] Peterson VM , Zhang KX , Kumar N , Wong J , Li L , Wilson DC , et al. Multiplexed quantification of proteins and transcripts in single cells. Nat Biotechnol. 2017;35:936–9. 28854175 10.1038/nbt.3973

[191] Lee J , Hyeon DY , Hwang D . Single‐cell multiomics: technologies and data analysis methods. Exp Mol Med. 2020;52:1428–42. 32929225 10.1038/s12276-020-0420-2 PMC8080692

[192] Burgess DJ . Spatial transcriptomics coming of age. Nat Rev Genet. 2019;20:317–7. 30980030 10.1038/s41576-019-0129-z

[193] Chen H , Murray E , Sinha A , Laumas A , Li J , Lesman D , et al. Dissecting mammalian spermatogenesis using spatial transcriptomics. Cell Rep. 2021;37:109915. 34731600 10.1016/j.celrep.2021.109915 PMC8606188

